# High‐Performance Lithium‐Oxygen Battery Electrolyte Derived from Optimum Combination of Solvent and Lithium Salt

**DOI:** 10.1002/advs.201700235

**Published:** 2017-07-25

**Authors:** Su Mi Ahn, Jungdon Suk, Do Youb Kim, Yongku Kang, Hwan Kyu Kim, Dong Wook Kim

**Affiliations:** ^1^ Advanced Materials Division Korea Research Institute of Chemical Technology 141 Gajeong‐ro Yuseong‐gu Daejeon 34114 South Korea; ^2^ Global GET‐Future Laboratory and Department of Advanced Materials Chemistry Korea University 2511 Sejong‐ro Jochiwon Sejong 30019 South Korea

**Keywords:** lithium nitrate, lithium oxygen batteries, NO_2_^−^/NO_2_ redox reaction, tetramethylene sulfone

## Abstract

To fabricate a sustainable lithium‐oxygen (Li‐O_2_) battery, it is crucial to identify an optimum electrolyte. Herein, it is found that tetramethylene sulfone (TMS) and lithium nitrate (LiNO_3_) form the optimum electrolyte, which greatly reduces the overpotential at charge, exhibits superior oxygen efficiency, and allows stable cycling for 100 cycles. Linear sweep voltammetry (LSV) and differential electrochemical mass spectrometry (DEMS) analyses reveal that neat TMS is stable to oxidative decomposition and exhibit good compatibility with a lithium metal. But, when TMS is combined with typical lithium salts, its performance is far from satisfactory. However, the TMS electrolyte containing LiNO_3_ exhibits a very low overpotential, which minimizes the side reactions and shows high oxygen efficiency. LSV‐DEMS study confirms that the TMS‐LiNO_3_ electrolyte efficiently produces NO_2_^−^, which initiates a redox shuttle reaction. Interestingly, this NO_2_^−^/NO_2_ redox reaction derived from the LiNO_3_ salt is not very effective in solvents other than TMS. Compared with other common Li‐O_2_ solvents, TMS seems optimum solvent for the efficient use of LiNO_3_ salt. Good compatibility with lithium metal, high dielectric constant, and low donicity of TMS are considered to be highly favorable to an efficient NO_2_^−^/NO_2_ redox reaction, which results in a high‐performance Li‐O_2_ battery.

## 1 Introduction

The lithium‐oxygen (Li‐O_2_) battery is a promising energy storage system that is particularly adaptable to electric vehicles, primarily because it can deliver much higher specific energy than the current lithium‐ion battery.^1^, ^2^, ^3^, ^4^, ^5^ However, realizing the commercial potential of the Li‐O_2_ battery has been stymied by several critical issues such as high overpotential at charge, low cycling reversibility, and high capacity fading during cycling.^6^, ^7^, ^8^, ^9^ These drawbacks result primarily from the parasitic decomposition of the cell components under the harsh operating conditions of the Li‐O_2_ cell. The carbon cathode is prone to an oxidative attack by active species at high overpotential to produce parasitic gas CO_2_, resulting in poor rechargeability in the cell.^5^ The stability issue of lithium metal anode is also critical for stable cycling of the cell.^9^ However, among the components the battery electrolyte is most susceptible to deterioration, because it is in direct contact with all of the Li‐O_2_ cell components. The cell electrolyte is exposed to nucleophilic attack by the reactive reduced products (i.e., O_2_^−^, LiO_2_, and Li_2_O_2_) present on the cathode during the discharge process, and is vulnerable to reactive lithium metal on the anode during cycling. The by‐products from the electrolyte decomposition produce a significant increase in the cell voltage during the charging process, which promotes oxidative decomposition of the electrolyte. Repeated cycling of the cell produces side reactions, and the resulting parasitic products accumulate on the cathode, which eventually results in cell failure after a brief cycle life.^10^, ^11^, ^12^ Consequently, the commercial future of a sustainable and rechargeable Li‐O_2_ battery rests on identifying and developing a highly stable and optimized electrolyte.

Lithium battery electrolytes generally comprise aprotic, organic solvents, and lithium salts. The organic solvents must have several desirable properties including oxidative stability, compatibility with lithium metal, low volatility, and high solubility for lithium salt. Of these properties, stability to oxidative decomposition and compatibility with lithium metal are of the utmost importance for stable operation in the Li‐O_2_ battery chemistry. In this work, tetramethylene sulfone (TMS) was found to afford much higher stability than other typical Li‐O_2_ solvents such as dimethylacetamide (DMA), dimethyl sulfoxide (DMSO), and tetraethylene glycol dimethylether (TEGDME). In spite of its stability, however, the cycle performance of the Li‐O_2_ cell employing a TMS‐based electrolyte was far from satisfactory. Other studies have reported that TMS electrolytes exhibited a high charge potential above 4 V, low capacity retention on cycling, and rapid capacity fading over a short cycle life.^13^, ^14^ Examination of the cell components using X‐ray diffraction, infrared spectroscopy, and mass spectrometry revealed that the TMS electrolytes decomposed to form a large quantity of by‐products, which accumulated on the cathode. This result implied that a stable solvent is not, in and of itself, sufficient to sustain efficient cycling in a Li‐O_2_ battery.

In several recent literature reports, when the lithium nitrate (LiNO_3_) salt was included in the electrolyte in a Li‐O_2_ cell, the cell exhibited better cycle performance than when other lithium salts were used, including lithium bis(trifluoromethane) sulfonimide (LiTFSI).^15^, ^16^, ^17^, ^18^, ^19^, ^20^, ^21^, ^22^, ^23^, ^24^, ^25^, ^26^, ^27^ Walker et al.^16^ demonstrated that use of LiNO_3_ in place of LiTFSI resulted in the production of a stable solid‐electrolyte interphase (SEI) on the negative electrode that protected the labile DMA solvent from the lithium metal anode and the stable Li‐O_2_ cell successfully executed more than 80 cycles. Sharon et al.^17^ reported the beneficial effects of using LiNO_3_ in a diglyme solvent to form an electrolyte that exhibited lower charging overpotential than a similar electrolyte containing LiTFSI. In our recent work,^20^, ^21^, ^22^ we also confirmed that amide solvents together with select lithium salts improved the stability of the solvent, reduced the cell's charging overpotential, and enhanced oxygen evolution efficiency during charging in comparison with other lithium salts.

Encouraged by these results, in this current work TMS was combined with LiNO_3_ salt and the performance of the resulting electrolyte was investigated in detail. The new electrolyte was subjected to differential electrochemical mass spectrometry (DEMS) analysis, which showed that a Li‐O_2_ cell charged with this electrolyte exhibited far superior performance than similar TMS‐based electrolytes containing other lithium salts. In an extended study, other typical solvents such as DMA, DMSO, and TEGDME were also combined with LiNO_3_ in a comparative study of their performance. Interestingly, the experimental results indicated that the performance of the salt is strongly affected by the solvent used to prepare the electrolyte.

The main objectives of this study are to determine why the LiNO_3_ salt promoted improved performance in Li‐O_2_ and to identify the reasons why the improved effect of the salt is strongly dependent on the solvent. Employing linear sweep voltammetry (LSV) combined with DEMS analysis, the formation of NO_2_^−^ was quantified and the oxidation potentials of the redox mediators, NO_2_^−^ to NO_2_, were determined. The formation of NO_2_^−^ and the subsequent NO_2_^−^/NO_2_ redox shuttle reactions were found to be greatly dependent on the solvent properties including stability, dielectric constant, and donicity. By comparing all of the quantitative DEMS results of the various electrolytes, the solvent that exhibited better performance with LiNO_3_ was identified. Consequently, these results may provide a suitable guide for the identification of an optimum electrolyte for the high‐performance Li‐O_2_ battery.

## 2 Results and Discussion

### 2.1 Stability of Solvents

**Figure 1:**
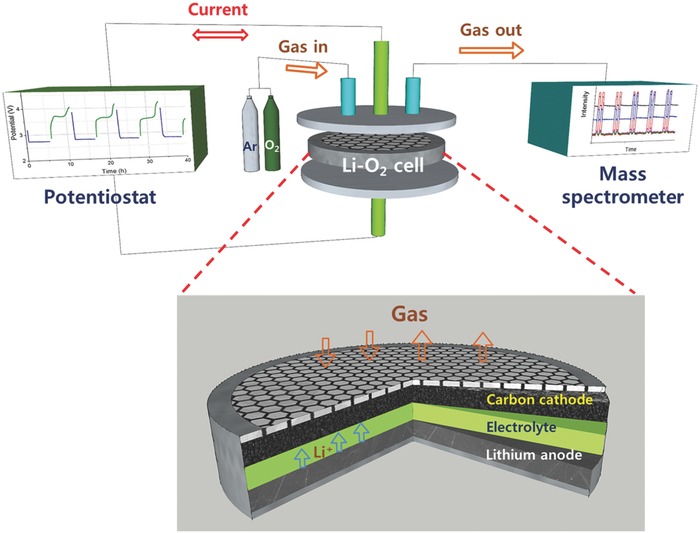
Schematic illustration of in situ DEMS setup and coin‐type Li‐O_2_ cell used for this work.

**Figure** **1** schematically illustrates in situ DEMS setup and coin‐type Li‐O_2_ cell used for this work. In order to compare the oxidative stability of the solvents, the Li‐O_2_ cells were prepared by assembling all the cell components after they had been individually saturated with each solvent containing 1 m LiTFSI. The LSV‐DEMS analysis was conducted on the pristine Li‐O_2_ cells under an argon atmosphere subjected to a voltage sweep from open‐circuit voltage (OCV) to 5.0 V to measure the change in the anodic (oxidative) current as well as the evolution of CO_2_ and H_2_ gases due to any solvent decomposition reactions.

**Figure 2:**
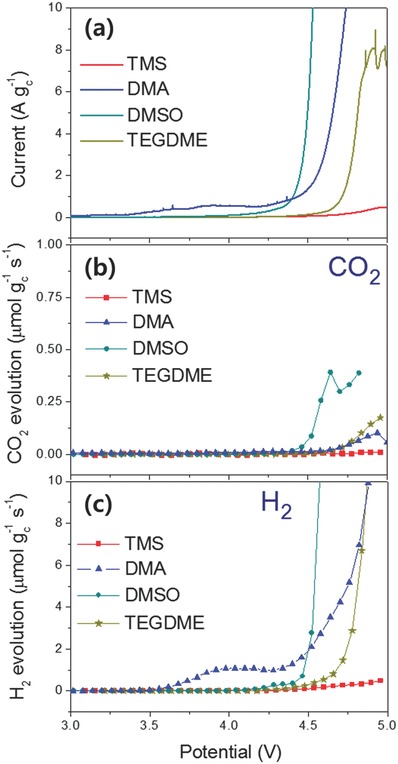
a) Anodic current, b) CO_2_ evolution rate, and c) H_2_ evolution rate measured by LSV‐DEMS analysis during linear oxidative scan from OCV to 5 V at a scan rate of 0.1 mV s^−1^, conducted using pristine Li‐O_2_ cells employing various solvents, including TMS, DMA, DMSO, and TEGDME containing 1 m LiTFSI under an argon atmosphere.

**Figure** **2** shows results of LSV‐DEMS analysis, which suggest that the TMS possessed superior oxidative stability and was more stable to lithium metal than other typical Li‐O_2_ electrolyte solvents such as DMA, DMSO, and TEGDME. Oxidative decomposition of the solvents occurs primarily at high potentials during the charging process in the Li‐O_2_ cells.^28^ This is why the solvent used in the Li‐O_2_ cells should have a wide electrochemical voltage window to ensure good oxidative stability at high potentials. Figure 2a shows the profiles of the anodic current generated during the LSV scans for the individual Li‐O_2_ cells containing TMS, DMA, DMSO, and TEGDME electrolytes. As can be seen, the Li‐O_2_ cell with the DMA generated a significant amount of anodic current even at potentials of 3.35 V, followed by a substantial increase in the current from 4.5 V. The DMSO‐LiTFSI Li‐O_2_ cell exhibited a gradual increase in anodic current from 4.0 V and then exhibited an abrupt increase at 4.4 V, indicating that a considerable amount of oxidative decomposition occurred in the electrolyte at this potential. Although the Li‐O_2_ cell with the TEGDME electrolyte displayed better stability as evidenced by the small amount of anodic current generated to 4.5 V, after reaching 4.7 V the cell exhibited a significant increase in the anodic current, indicating that some electrolyte decomposition had occurred in the cell at that potential. Contrary to the other solvents, no detectable current was observed in the cell containing the TMS electrolyte until the cell reached 4.7 V and even after this potential was attained, only a small amount of current was detected, which was indicative of the better oxidative stability of the TMS electrolyte. This result agreed well with the experimental results presented in a recent report,^13^ in which TMS exhibited better stability at an oxidation potential of 5.6 V in comparison to other common Li‐O_2_ solvents such as DMSO (4.8 V), TEGDME (5.3 V), and dimethyl formamide (DMF, 5.1 V). Bardé et al.^14^ also reported a similar result where the anodic stability of TMS was much higher than DMSO (5.2 V for TMS and 4.2 V for DMSO) as determined by voltammetric analysis.

Evolution of CO_2_ gas, which is a typical parasitic gas that results from the oxidative decomposition of the solvents, was assessed for the various test cells during the LSV scan, and the results are presented in Figure 2b. As can be seen in this figure, the cell containing the DMSO‐based electrolyte generated CO_2_ gas beginning at a cell voltage of 4.4 V, which was the lowest onset potential of all the solvents. At 4.7 V, DMA and TEGDME exhibited a higher onset potential than DMSO. In the Li‐O_2_ cells containing TMS, a negligible amount of CO_2_ gas was detected even up to a cell voltage of 5.0 V. Based on the results of the highest onset potential for the generation of anodic current and CO_2_ evolution, together with the results for the amount of gas generated during the LSV scan to 5.0 V, it was concluded that TMS possessed the highest oxidative stability of the solvents tested.

Figure 2c shows the evolution behavior of H_2_, another parasitic reaction gas, during the LSV testing. In the case of DMA, H_2_ gas began being evolved at 3.5 V, which is the lowest onset potential of all the solvents, and after this potential was reached, a significant increase in the rate of H_2_ gas evolution was observed at 4.4 V. A possible source for the evolution of H_2_ gas in the cell may be found in the reductive decomposition of the solvents as a result of their reaction with the lithium metal anode.^16^ When 1 m LiNO_3_ was used as a supporting salt in place of 1 m LiTFSI, the DMA‐LiNO_3_ electrolyte exhibited a much higher onset potential for H_2_ gas evolution than the DMA‐LiTFSI electrolyte as shown in Figure S1b (Supporting Information). This was interpreted to mean that a surface layer had been formed on the lithium metal by its reaction with the LiNO_3_ salt, which passivated the lithium prohibiting the DMA solvent from contact with the native lithium metal as suggested in the previous publications.^16^, ^17^ The substantial decrease of H_2_ gas with the addition of LiNO_3_ suggested that the reactive lithium metal was a possible cause for the parasitic H_2_ gas evolution. It is noteworthy that the evolution profile of the H_2_ gas closely resembled the profile of the anodic current during the LSV potential scan. When the lithium salt in the electrolyte was changed from LiTFSI to LiNO_3_ in DMA, the evolution of H_2_ gas was suppressed and the onset potential for gas evolution shifted from 3.5 to 4.0 V (Figure S1b, Supporting Information). A similar result was observed in the profile of the anodic current with the onset potential shifting from 3.3 to 4.0 V (Figure S1a, Supporting Information). Replacement of the lithium metal with partially charged lithium iron phosphate (LFP) as an anode in the Li‐O_2_ cells containing the TEGDME‐LiTFSI electrolyte confirmed that the pure lithium metal may react with the electrolyte solvents resulting in the evolution of H_2_ gas (Figure S2, Supporting Information). The H_2_ gas evolution was substantially subdued up to 4.8 V with the LFP as the anode in the cell, whereas the Li‐O_2_ cell with pure lithium metal anode generated a considerable amount of H_2_ gas beginning at 4.4 V. Therefore, it appeared that the stability of the solvents to lithium metal could be estimated by observing the onset potential and amount of H_2_ gas evolved during LSV‐DEMS analysis. From this perspective, TMS was considered to be far more compatible with lithium metal than the other solvents, because this solvent showed the highest onset potential and the lowest amount of H_2_ evolved as depicted in Figure 2c. In contrast, Figure 2c indicates that DMA exhibited the lowest stability to lithium metal based on its lowest onset potential for H_2_ evolution. In addition, H_2_ gas began to evolve at 4.2 V for DMSO and 4.4 V for TEGDME, implying that DMSO was less stable to lithium metal than TEGDME. Overall, the LSV‐DEMS results in Figure 2 suggested that TMS had far better oxidative stability and was more stable to lithium metal.

### 2.2 Effect of Lithium Salts

**Figure 3:**
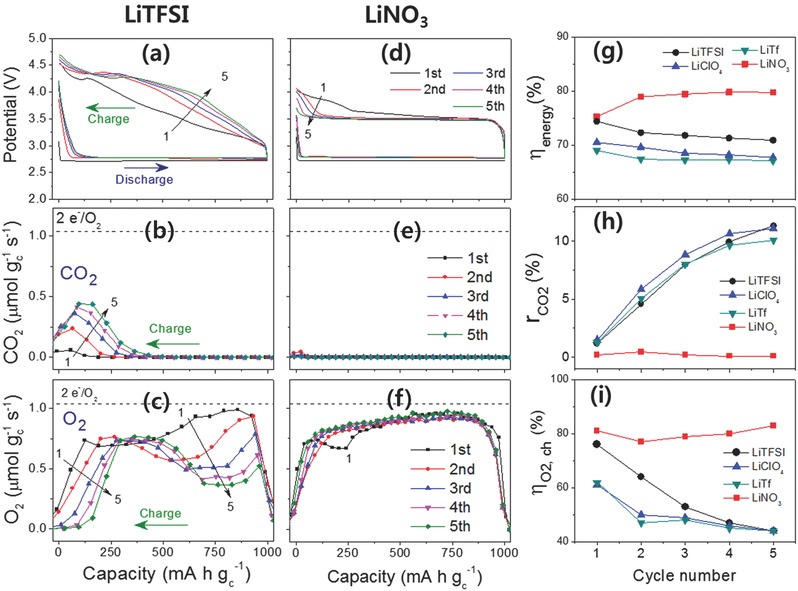
a,d) Potential profiles, b,e) CO_2_ evolution rate at charge, and c,f) O_2_ evolution rate at charge in Li‐O_2_ cells with TMS electrolytes containing a–c) 1 m LiTFSI and d–f) 1 m LiNO_3_ during the five‐cycle test. g) Energy efficiency, h) CO_2_ evolution ratio, and i) O_2_ efficiency during charge in the Li‐O_2_ cells with separate TMS electrolytes containing 1 m LiTFSI, LiClO_4_, LiTf , and LiNO_3_ during the five‐cycle test. The dashed lines labeled as 2 e^−^/O_2_ in (b,c,e,f) indicate the O_2_ evolution rate following the ideal reaction 2Li^+^ + O_2_ + 2e^−^ ↔ Li_2_O_2_.

The TMS electrolyte was prepared by dissolving a typical lithium salt, LiTFSI, in TMS and the performance of the resulting Li‐O_2_ cells was investigated using in situ DEMS as shown in **Figure** **3**a–c. In spite of the high stability of the solvent, the cycle performance of the TMS‐LiTFSI electrolyte was disappointing. The electrolyte exhibited a considerable amount of CO_2_ gas evolution during charging of the cell, which was indicative of oxidative parasitic reactions. The CO_2_ gas ratio (r_CO2_) in the first cycle was not very high (1.2%), but the value greatly increased from the second to the fifth cycles, eventually reaching more than 11%. This implied that additional oxidative decomposition reactions were occurring as the cell was cycled (Figure 3b,h). The degree of parasitic reaction will inevitably affect the oxygen evolution reaction during charging, which will adversely affect the reversibility of the cell. As evidence of this, the charge oxygen efficiency (η_O2_, _ch_) was greatly decreased from 76% in the first cycle to a mere 44% in the fifth cycle (Figure 3c,i). Cycling of the TMS‐LiTFSI Li‐O_2_ cell was terminated after only nine cycles as a result of the accumulation of parasitic reactions as shown in Figure S3 (Supporting Information).

**Figure 4:**
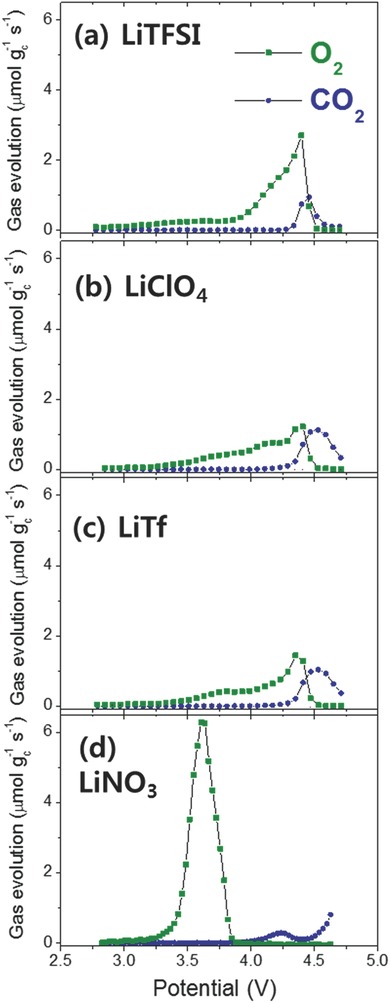
Evolution rate of O_2_ and CO_2_ measured by LSV‐DEMS analysis during linear oxidative scans from OCV to 4.7 V at 0.1 mV s^−1^, conducted on Li_2_O_2_‐loaded Li‐O_2_ cells using various TMS electrolytes containing a) LiTFSI, b) LiClO_4_, c) LiTf, and d) LiNO_3_. Li_2_O_2_ was deposited in situ on the cathode of the Li‐O_2_ cells by performing two discharge–charge cycles and a third discharge to a cell capacity of 1000 mA h g_c_^−1^ (see the Supporting Information for details).

**Figure** **4**a shows the LSV‐DEMS analysis of the TMS‐LiTFSI Li‐O_2_ cell loaded with Li_2_O_2_ that was produced by performing two discharge–charge cycles and a third discharge process to a cell discharge capacity of 1000 mA h g_c_^−1^. The LSV‐DEMS analysis monitored the evolution of O_2_ gas, which resulted from the oxidation of Li_2_O_2_, and the generation of CO_2_ gas, which was due to the oxidative decomposition of the parasitic reaction products during the oxidative scan from OCV to 4.7 V. This LSV‐DEMS study of the cell containing deposited Li_2_O_2_ provided information of the onset potential and peak potential, where the evolution of O_2_ and CO_2_ gases began and reached their maximum, as reported in our previous publications.^20^, ^21^ Although O_2_ gas evolved at 3.2 V as shown in Figure 4a, only a small amount was generated. A substantial amount of O_2_ evolution occurred from 3.8 to 4.5 V with a maximum value at 4.35 V. The CO_2_ gas was detected from 4.3 to 4.6 V with a peak value at 4.45 V, which resulted in a large overlap in the gas evolution potential regions of these two gases (O_2_ and CO_2_). In other words, the oxidation of Li_2_O_2_ (namely, O_2_ gas evolution) was inevitably accompanied by a parasitic reaction and evolution of CO_2_ gas, which occurred from 4.3 to 4.6 V. When the CO_2_ gas evolution profile in the Li_2_O_2_‐loaded Li‐O_2_ cell (Figure 4a) was compared to that of the pristine Li‐O_2_ cell (Figure 2b), the CO_2_ gas evolved at 4.3 to 4.6 V appeared to be associated with a parasitic reaction induced by Li_2_O_2_, because this evolution of CO_2_ did not occur in the same potential region in the pristine Li‐O_2_ cell without any Li_2_O_2_.

The TMS electrolytes with other lithium salts such as LiClO_4_ and LiTf also showed poor cycle performance as depicted in Figure S4 (Supporting Information). Actually, there was a kind of similarity in the cycle performance of the TMS electrolytes containing LiTFSI, LiClO_4_, and LiTf. As the various Li‐O_2_ cells with these three lithium salts proceeded from the first to the fifth cycle, the charge potential increased (and hence the energy efficiency decreased by approximately 3%), the oxygen efficiency during charge deteriorated by ≈30% for LiTFSI, and ≈20% for LiClO_4_ and LiTf, and the CO_2_ gas evolution increased by ≈10%. Similar amounts and trends were observed in the evolution of CO_2_ gas during five‐cell cycles (Figure 3b,h and Figure S4b,e, Supporting Information) and the same onset potential of 4.3 V for CO_2_ generation was observed in the LSV‐DEMS analysis of these lithium salts (Figure 4a–c), suggesting that similar parasitic reactions occurred in the TMS electrolytes containing LiTFSI, LiClO_4_, and LiTf.

When LiNO_3_ was used as the lithium salt in the TMS electrolyte, a strikingly different performance resulted both in terms of the charge overpotential and the evolution of O_2_ and CO_2_ gases. A noticeably flat voltage plateau was observed for the cell from the beginning to the end of the charge process where the cell held 3.5 V during five cycles as shown in Figure 3d. This apparently different charge profile in the TMS electrolyte containing LiNO_3_ indicated that a different mechanism was responsible for the oxidation of Li_2_O_2_ (this will be discussed later). This low overpotential during cell charging in TMS‐LiNO_3_ electrolyte enhanced the cell's energy efficiency during cycling to 80%, which was about 10% higher than that of the TMS electrolytes with other lithium salts (Figure 3g). Along with the low overpotential at charge, the parasitic decomposition reaction was considerably suppressed so that evolution of CO_2_ gas was greatly reduced as shown in Figures 3e,h. Compared with the other TMS electrolytes that generated a large amount of CO_2_ gas, the TMS‐LiNO_3_ electrolyte evolved a negligible amount of CO_2_ gas during the five cycles. Moreover, the TMS‐LiNO_3_ electrolyte displayed a much higher charge oxygen efficiency of over 80% (81% in the first cycle and 83% in the fifth cycle). It is noteworthy that the TMS‐LiNO_3_ electrolyte exhibited a stable evolution of oxygen during the whole charge process and during the five cycles. Oxygen gas was evolved in nearly similar quantities from the start to the end in the charge process in the TMS‐LiNO_3_ electrolyte as shown in Figure 3f. However, the evolution of O_2_ fluctuated during the charge process in the TMS electrolytes prepared with lithium salts other than LiNO_3_ (Figure 3c and Figure S4c,f, Supporting Information). In addition, the TMS‐LiNO_3_ electrolyte maintained stable oxygen efficiency from the first to the fifth cycle, contrary to the other TMS electrolytes that exhibited a steep decrease in the oxygen efficiency on cycling (Figure 3i).

When the evolution patterns and potentials of the O_2_ and CO_2_ gases of the LSV‐DEMS results are compared in Figure 4, it is again apparent that the TMS‐LiNO_3_ electrolyte was far different from the other TMS electrolytes. The LSV‐DEMS of the Li_2_O_2_‐loaded Li‐O_2_ cell with the TMS‐LiNO_3_ electrolyte showed that O_2_ evolved from 3.3 to 3.8 V with a peak value at 3.55 V. The O_2_ evolution in such a low potential region was completely isolated from CO_2_ gas evolution, which began at 4.0 V (Figure 4d). This meant that a very low amount of CO_2_ gas was evolved during the cycling in the TMS‐LiNO_3_ electrolyte cell as shown in Figure 3e, which was attributed to the lower charge overpotential for O_2_ gas evolution (namely, oxidation of Li_2_O_2_). In other words, the lower charge overpotential in the TMS‐LiNO_3_ electrolyte was the key factor in preventing a parasitic reaction and suppression of CO_2_ gas evolution in that Li‐O_2_ cell.

### 2.3 NO_2_^−^/NO_2_ Redox Reaction Derived from LiNO_3_

The question of what makes the TMS‐LiNO_3_ electrolyte exhibit such a low potential for oxidation of Li_2_O_2_ will be addressed next. A LSV‐DEMS investigation of the pristine Li‐O_2_ cell (without loaded Li_2_O_2_) with the TMS‐LiNO_3_ electrolyte confirmed that NO_2_^−^ existed in the electrolyte and it was oxidized to NO_2_ during the oxidative scan. Three kinds of Li‐O_2_ cells each with a different TMS electrolyte were tested. These electrolytes separately contained (a) 1 m LiTFSI and 0.02 m NaNO_2_, (b) 1 m LiTFSI, and (c) 1 m LiNO_3_ (Figure S5, Supporting Information). The three individual cells were scanned from OCV to 4.5 V while monitoring the anodic current and NO_2_ gas evolution using DEMS. In the TMS‐LiTFSI electrolyte with 0.02 m NaNO_2_, a noticeable amount of oxidative current and NO_2_ gas was observed in the potential range between 3.5 and 3.7 V as shown in Figure S5a (Supporting Information). However, there was no detectable current or NO_2_ gas in the TMS‐LiTFSI electrolyte in the same potential region (Figure S5b, Supporting Information). It was believed that NO_2_^−^ in the TMS‐LiTFSI electrolyte with NaNO_2_ was oxidized to NO_2_ during the LSV scan and then it was evolved as a gas. NO_2_ is capable of oxidizing Li_2_O_2_ to produce Li^+^ and O_2_ when it is reduced to NO_2_^−^ as verified in a recent report by Uddin et al.^19^ who described the reaction of 2NO_2_ + Li_2_O_2_ → 2NO_2_^−^ + 2Li^+^ + O_2_. It would appear then that NO_2_ played the role of redox mediator for the oxidation of Li_2_O_2_, which resulted in the evolution of O_2_ gas in the potential of the NO_2_^−^/NO_2_ redox reaction. To confirm this speculation, a Li‐O_2_ cell that contained the TMS‐LiTFSI electrolyte with NaNO_2_ was prepared and then Li_2_O_2_ was deposited on the cathode using the previously described discharge process to a cell capacity of 1000 mA h g_c_^−1^. As shown in Figure S6a (Supporting Information), the LSV‐DEMS analysis of this cell revealed that O_2_ gas was indeed generated in a similar potential region to that of NO_2_ gas evolution as shown in Figure S5a (Supporting Information). When this result was compared with the O_2_ evolution profile of the TMS‐LiTFSI electrolyte in Figure 4a, the potential for O_2_ evolution (namely, oxidation of Li_2_O_2_) was significantly lowered by the addition of 0.02 m NaNO_2_ as shown in Figure S6a (Supporting Information). The peak potential, at which a maximum amount of O_2_ is evolved, was shifted from 4.45 V (for TMS‐LiTFSI, Figure 4a) to 3.5 V (for TMS‐LiTFSI with 0.02 m NaNO_2_; Figure S6a, Supporting Information). As a result, the charge overpotential was also considerably reduced by the addition of 0.02 m NaNO_2_ as shown in Figure S6b (Supporting Information). There was an evident flat plateau at 3.5 V in the charging of Li‐O_2_ cell containing the TMS‐LiTFSI electrolyte with NaNO_2_, whereas the TMS‐LiTFSI electrolyte alone showed a much higher overpotential during charge as shown in Figure 3a.

In Figure S5c (Supporting Information), it can be seen that TMS‐LiNO_3_ electrolyte exhibited an anodic current and NO_2_ gas evolution in the same potential region of 3.5–3.7 V as that of TMS‐LiTFSI with 0.02 m NaNO_2_. These LSV‐DEMS results appeared to indicate that NO_2_^−^ was present in the TMS‐LiNO_3_ Li‐O_2_ cell and it could act as a redox mediator as was the case in the TMS‐LiTFSI with NaNO_2_. The NO_2_^−^/NO_2_ redox shuttle reaction was believed to be the reason why the TMS‐LiNO_3_ electrolyte exhibited a low potential of 3.55 V for the oxidation of Li_2_O_2_ (Figure 4d), and hence, a highly suppressed overpotential at charge (Figure 3d). When the amount of NO_2_ gas evolved in the TMS‐LiNO_3_ electrolyte was compared with that from TMS‐LiTFSI containing 0.02 m NaNO_2_ (0.70 µmol for TMS‐LiNO_3_ and 1.23 µmol for TMS‐LiTFSI with 0.02 m NaNO_2_), the concentration of NO_2_^−^ contained in the TMS‐LiNO_3_ Li‐O_2_ cell was estimated to be 0.011 m, assuming that the amount of evolved NO_2_ gas was proportional to the concentration of NO_2_^−^ ((0.70/1.23) × 0.02 m = 0.011 m).

### 2.4 Effect of Solvents on NO_2_^−^/NO_2_ Redox Reaction

**Figure 5:**
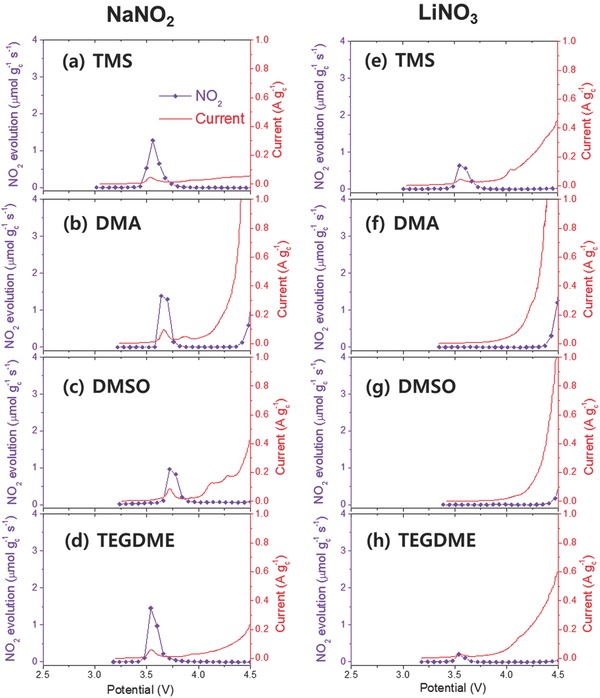
NO_2_ evolution rate and anodic current measured by LSV‐DEMS analysis of a linear oxidative scan from OCV to 4.5 V at 0.1 mV s^−1^ conducted on Li‐O_2_ cells with a) TMS, b) DMA, c) DMSO, and d) TEGDME containing 1 m LiTFSI and 0.02 m NaNO_2_ (except for DMA), and e) TMS, f) DMA, g) DMSO, and h) TEGDME containing 1 × 10^−3^ m LiNO_3_ (except for TEGDME). 1 m LiNO_3_ and 0.02 m NaNO_2_ were dissolved in DMA in the case of curve (b), because DMA with LiTFSI and NaNO_2_ suffered from severe oxidative decomposition during the LSV scan as shown in Figure S7 (Supporting Information), and hence, the detection of NO_2_ gas was failed. Due to limited solubility, 0.5 m LiNO_3_ was dissolved in TEGDME for curve (h).

The next question to be answered is whether the LiNO_3_ salt is also as effective in other solvents such as DMA, DMSO, and TEGDME. The LSV‐DEMS data shown in **Figure** **5** clearly show that the evolution of NO_2_ gas and NO_2_^−^/NO_2_ redox reaction were not as effective in the other solvents containing LiNO_3_. In the DMA and DMSO electrolytes containing LiNO_3_, no NO_2_ gas or anodic current was detected in the potential range of 3.5–3.8 V in the LSV‐DEMS scans (Figure 5f,g). It should be noted that all of the solvents containing 0.02 m NaNO_2_ generated NO_2_ gas and exhibited an anodic current during the LSV scans and the amount of the gas was not much different in any of the solvents as shown in Figure 5a–d (1.23, 1.29, 1.10, and 1.25 µmol for TMS, DMA, DMSO, and TEGDME with 0.02 m NaNO_2_, respectively). This result indicated that the quantity of NO_2_ gas evolved was dependent on the concentration of NO_2_^−^ in the electrolyte. Therefore, no detection of NO_2_ gas in DMA‐LiNO_3_ and DMSO‐LiNO_3_ electrolytes meant that NO_2_^−^ was not present in the electrolytes and the NO_2_^−^/NO_2_ redox shuttle reaction would not work during the charging process.

**Figure 6:**
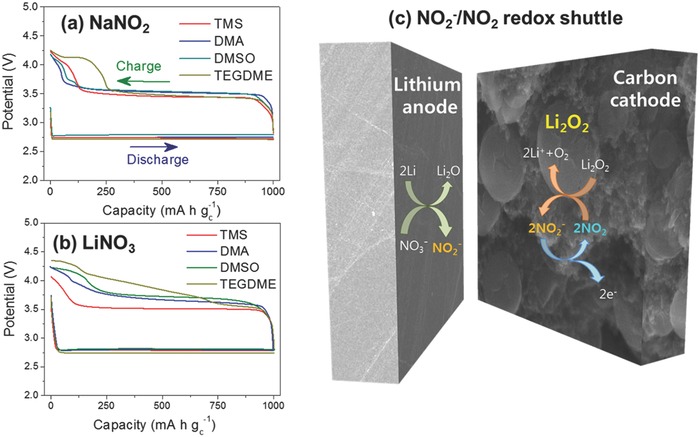
a) Potential profiles of TMS, DMSO, and TEGDME electrolytes containing 1 m LiTFSI and 0.02 m NaNO_2_, and DMA electrolyte containing 1 m LiNO_3_ and 0.02 m NaNO_2._during the first cycle. b) Potential profiles of TMS, DMA, DMSO, and TEGDME electrolytes containing LiNO_3_ during the second cycle, which was obtained from Figures 3d and 7a,d,g. c) Schematic illustration of the formation of NO_2_^−^ from the reaction of LiNO_3_ on the surface of lithium anode, oxidation of NO_2_^−^ on the carbon cathode, and reduction of NO_2_ with the reaction of Li_2_O_2_ to produce O_2_.

The voltage profiles of the solvents with (a) 1 m LiTFSI and 0.02 m NaNO_2_ (except for DMA with 1 m LiNO_3_ and 0.02 m NaNO_2_) and (b) 1 m LiNO_3_ (except for TEGDME with 0.5 m LiNO_3_) are compared in **Figure** **6**a,b. The TMS‐LiTFSI with NaNO_2_ exhibited a flat plateau in the potential of around 3.5 V during the charging process, which seemed to indicate that oxidation of Li_2_O_2_ via NO_2_^−^/NO_2_ redox shuttle reaction occurred. A similar profile was also observed in TMS‐LiNO_3_ electrolyte, possibly due to the same redox reaction. However, the DMA and DMSO electrolytes containing NaNO_2_ displayed a charge plateau at 3.55 V, whereas the electrolytes with LiNO_3_ exhibited a charge profile at much higher potential of about 3.7 V. It is believed that this difference occurred, because the NO_2_^−^/NO_2_ redox shuttle reaction was not working during the charging process in DMA or DMSO electrolytes containing LiNO_3_.

In the case of the TEGDME‐LiNO_3_ electrolyte, NO_2_ gas was detected in the potential region of 3.5–3.7 V (Figure 5h), which was similar to the results for the TEGDME‐LiTFSI with 0.02 m NaNO_2_ electrolyte (Figure 5d). However, the quantity of NO_2_ gas was much smaller than that in the TMS‐LiNO_3_ electrolyte (0.19 µmol for TEGDME‐LiNO_3_ and 0.70 µmol for TMS‐LiNO_3_). It seemed that such a low quantity of NO_2_ gas was not adequate for an efficient NO_2_^−^/NO_2_ redox shuttle reaction. The charge profile of TEGDME‐LiNO_3_ electrolyte in Figure 6b consisted of a short plateau region at 3.6 V from the beginning to the first quarter of the charge capacity, and a long slope region with a gradual potential increase from the second quarter to the end. This result could be interpreted to mean that in the short plateau region at 3.6 V the NO_2_^−^/NO_2_ redox shuttle reaction may have occurred, but after this region another mechanism for Li_2_O_2_ oxidation was activated and it became dominant. The concentration of NO_2_^−^ in the TEGDME‐LiNO_3_ electrolyte might not be adequate for the redox reaction to be maintained during the entire charge process. Hence, the redox reaction occurred in the early stage of the charge process, which was followed by another oxidation process for Li_2_O_2_ in the remaining charge period. For comparison, TEGDME‐LiTFSI with the NaNO_2_ electrolyte contained sufficient NO_2_^−^, and, as a result, it maintained a flat voltage plateau from the beginning to the end of the oxidation of Li_2_O_2_ as shown in Figure 6a.

### 2.5 Optimum Solvent for NO_2_^−^/NO_2_ Redox Reaction

It seemed that the formation of NO_2_^−^ in the solvents containing LiNO_3_ was greatly affected by the solvent's stability with the lithium metal anode. As previously mentioned and illustrated in Figure 6c, NO_2_^−^ can be produced by the reaction of LiNO_3_ with lithium metal and it acts as a redox mediator in the oxidation of Li_2_O_2_ following the sequential reactions of; (1) generation of NO_2_^−^ on the lithium metal anode: 2Li + LiNO_3_ → Li_2_O + LiNO_2_; (2) oxidation of NO_2_^−^ on the cathode: NO_2_^−^ → NO_2_ + e^−^; and (3) reduction of NO_2_ with the reaction of Li_2_O_2_: 2NO_2_ + Li_2_O_2_ → 2NO_2_^−^ + 2Li^+^ + O_2_. To generate NO_2_^−^, lithium metal must react with LiNO_3_ via the above reaction pathway (1). When the solvent has insufficient stability and the reaction of lithium metal with the solvent is dominant, the formation of NO_2_^−^ via pathway (1) might be greatly impeded. As discussed in Figure 2c, DMA and DMSO exhibited high reactivity with lithium metal, resulting in the evolution of a large amount of H_2_ gas. It appeared that the high reactivity of these solvents severely interfered with the formation of NO_2_^−^. It is also possible that after NO_2_^−^ was formed by the reaction of lithium metal and LiNO_3_, NO_2_^−^ was attacked or damaged by the reaction between the lithium metal and the solvent, and was transformed into other chemical species. However, the LSV‐DEMS results for the four types of solvents with 0.02 m NaNO_2_ (Figure 5a–d) showed that similar amounts of NO_2_ gas were evolved regardless of the solvent, which suggested that the possibility of this scenario is low.

In a recent study by Walker et al.^16^ dealing with the DMA‐LiNO_3_ electrolyte, anodic current was observed in the potential region of approximately 3.7 V during the LSV scan, which was claimed to be attributed to the oxidation of NO_2_^−^. In addition, the authors confirmed the existence of NO_2_^−^ in the DMA‐LiNO_3_ electrolyte using UV–visible spectroscopy, although the data were obtained after 40 days of reaction between the lithium metal and LiNO_3_.^19^ This implied that the formation of detectable amounts of NO_2_^−^ may take a long time to occur in the electrolyte. Even with several repetitions of the LSV‐DEMS experiments on the DMA‐LiNO_3_ electrolyte, we failed to observe any anodic current and NO_2_ gas in the select potential region. And in the UV–visible spectroscopy study, the absorption spectrum due to NO_2_^−^ was not observed in the DMA‐LiNO_3_ solution with lithium metal contained. It should be noted that although LiNO_3_ is not effective for the NO_2_^−^/NO_2_ redox shuttle reaction in the DMA solvent, the salt does enhance the stability of DMA for Li as evidenced by the data shown in Figure S1 (Supporting Information), which compares the DMA‐LiTFSI and DMA‐LiNO_3_ electrolytes. This higher stability may result from the SEI layer formed by the reaction of LiNO_3_ with the lithium metal. Aurbach et al. confirmed that insoluble Li*_x_*NO*_y_* species are formed on the surface of lithium metal upon reaction with LiNO_3_ in their detailed investigation performed using Fourier transform‐infrared (FT‐IR) and X‐ray photoelectron spectroscopy (XPS) with a specialized apparatus.^29^ In addition, this type of SEI layer has been reported to play a key role in enhancing the cycle performance in a lithium sulfur battery.^30^, ^31^

Compared to DMA and DMSO, TEGDME with LiNO_3_ produces NO_2_ gas due to its higher stability, but the quantity of the gas is much smaller than in TMS. It should be noted that TEGDME has much lower dielectric constant than the other solvents (the dielectric constants of TMS, DMA, DMSO, and TEGDME are 43.4, 37.8, 46.7, and 7.8, respectively),^32^ and the solvent has a limited solubility for the LiNO_3_ salt. Dielectric constant is known a rough measure of polarity of solvents,^33^ and hence TEGDME is considered a solvent with low polarity. Following a thorough drying process, the anhydrous TEGDME can only dissolve LiNO_3_ to produce a 0.5 m solution, in our investigation. It can be possible that such low polarity is not favored for the effective formation of NO_2_^−^ via the above reaction pathway (1) or limits solubility of NO_2_^−^ in TEGDME solvent.

In another interesting point, it should be noted that the potential for NO_2_ gas evolution during the LSV scans was dependent on the solvents as shown in Figure 5a–d (or see Figure S8, Supporting Information). The TMS with NaNO_2_ evolved NO_2_ gas in the potential region of 3.5–3.7 V with a maximum at 3.55 V, which was similar to TEGDME. In the case of DMA and DMSO, the NO_2_ gas evolution occurred at a higher potential; the maximum potential was 3.7 V for DMA and 3.75 V for DMSO. The different potentials for the gas evolution was believed to be closely related to the solvating power of the solvents, which can be expressed by Gutmann donor number.^34^ TMS and TEGDME have much lower donor number than DMA and DMSO (the donor numbers for TMS, TEGDME, DMA, and DMSO are 14.8, 16.6, 27.8, and 29.8 kcal mol^−1^, respectively).^34^ In solution, the NO_2_^−^ could interact with a cation (i.e., Li^+^) and the resulting agglomerates of Li^+^ and NO_2_^−^ would be solvated by the solvent molecules, which is strongly affected by the donicity of the solvent.^35^, ^36^ This type of phenomenon is typical in a solution containing dissolved salts. The higher the donicity of the solvent, the stronger the solvation of the solvent molecules to attract the ionic agglomerates. In DMA or DMSO solvents with a higher donor number, the agglomerates between Li^+^ and NO_2_^−^ would be strongly solvated by the solvent molecules, which would impede the oxidation reaction of NO_2_^−^ on the cathode surface and result in a high polarization. Therefore, the oxidation of NO_2_^−^ would occur at higher potentials during the LSV scan than in the case of TMS or TEGDME with a lower donicity. This would result in the evolution of NO_2_ gas at a higher oxidation potential in DMA and DMSO. This shift in the NO_2_ evolution potential would also influence the charge potential as shown in Figure 6a. The DMA and DMSO electrolytes with NaNO_2_ showed a higher charge potential than the TMS and TEGDME electrolytes. This indicated that the lower donicity of TMS was favorable to a lower overpotential in the charge process as a result of the NO_2_^−^/NO_2_ redox reaction. Overall, with high stability, high dielectric constant, and low donicity, TMS appeared to be the optimum solvent for an efficient NO_2_^−^/NO_2_ redox reaction derived from LiNO_3_.

### 2.6 High‐Performance Li‐O_2_ Electrolyte

**Figure 7:**
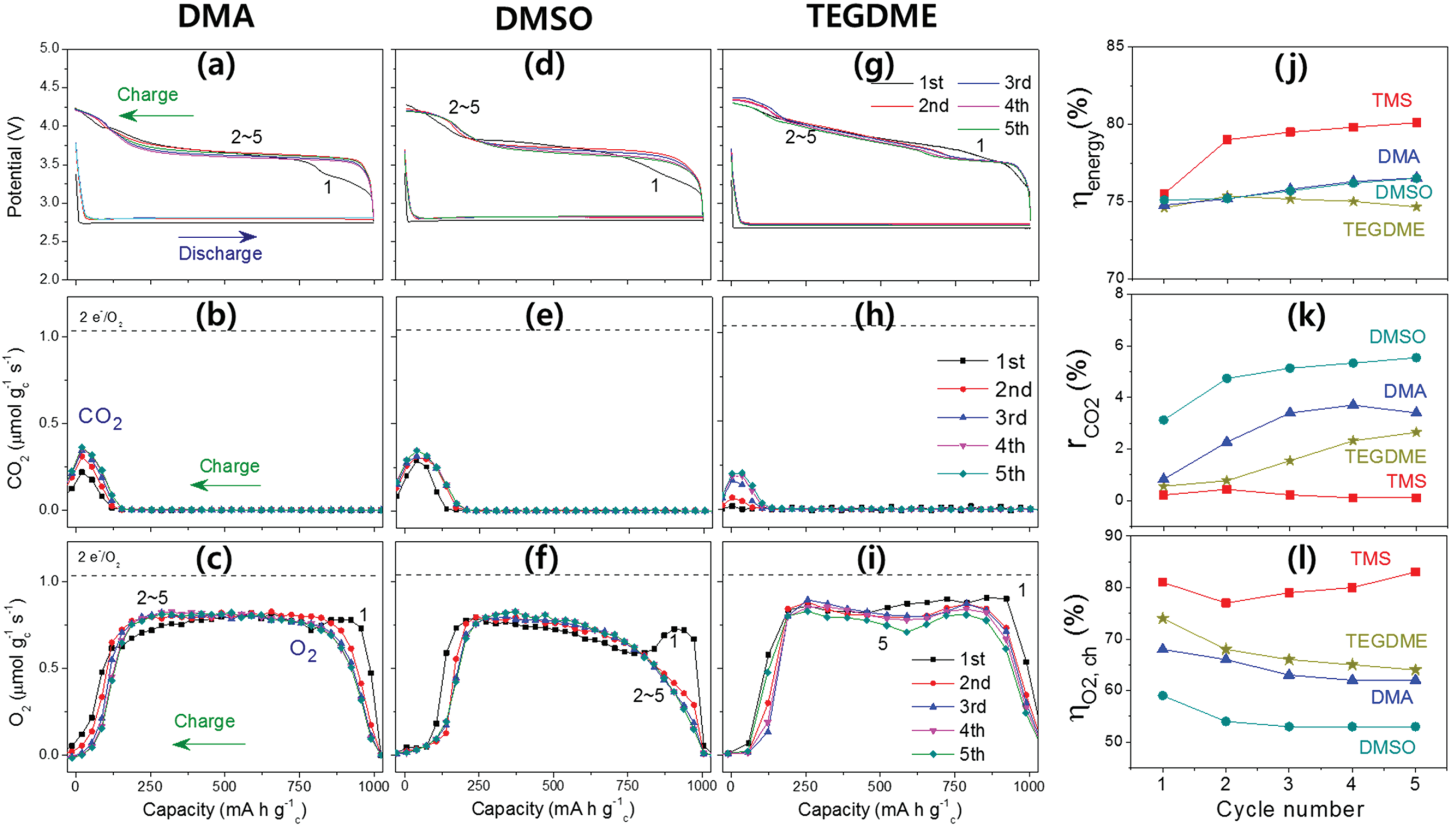
a,d,g) Potential profiles, b,e,h) CO_2_ evolution rate at charge, and c,f,i) O_2_ evolution rate at charge in Li‐O_2_ cells with (a,b,c) DMA, (d,e,f) DMSO, and (g,h,i) TEGDME electrolytes containing LiNO_3_ during the five‐cycle test. j) Energy efficiency, k) CO_2_ evolution ratio, and l) O_2_ efficiency during charge in the Li‐O_2_ cells with TMS, DMA, DMSO, and TEGDME electrolytes containing LiNO_3_ during the five‐cycle test.

As a result, the TMS‐LiNO_3_ electrolyte exhibited superior cycle performance in comparison to other solvents containing LiNO_3_. In Figure 3d–f and **Figure** **7**, the potential profiles and evolution of O_2_ and CO_2_ gases in the Li‐O_2_ cells with TMS, DMA, DMSO, and TEGDME electrolytes containing LiNO_3_ are shown during a five‐cycle test, which was performed using DEMS. Figure 7j–l compares the energy efficiency, CO_2_ ratio, and charge oxygen efficiency of the four electrolytes, and it can be seen that the performance of the TMS electrolyte was excellent. The first attribute of the TMS‐LiNO_3_ can be seen in the energy efficiency (Figure 7j). TMS electrolyte exhibited about 5% higher energy efficiency than other electrolytes. For example, the TMS electrolyte achieved 80.1% energy efficiency at the fifth cycle, while DMA, DMSO, and TEGDME electrolytes exhibited 76.5, 76.5, and 74.7%, respectively. This higher energy efficiency from the TMS‐LiNO_3_ electrolyte may have been due to efficient NO_2_^−^/NO_2_ redox shuttle reaction, which produced a lower overpotential during the charging process. The lower overpotential in the TMS electrolyte could minimize the oxidative decomposition side reactions and suppress CO_2_ gas evolution (Figure 7k). All of the other electrolytes exhibited a higher overpotential at charge and hence they suffered from severe parasitic reactions and CO_2_ gas evolution, especially in the final stages of the charging process. Contrary to the TMS‐LiNO_3_ electrolyte showing a negligible amount of CO_2_ gas evolution during the five cycles, the other electrolytes evolved a large amount of CO_2_ gas, and, moreover, the amount of the gas increased as the cycling progressed. In the DMA electrolyte, the CO_2_ gas ratio (*r*_CO2_) increased from 2.4% in the first cycle to 4.6% in the fifth cycle. In DMSO, the value increased from 3.1% in the first cycle to 5.5% in the fifth cycle. In TEGDME electrolyte, *r*_CO2_ increased from 0.6% in the first cycle to 2.6% in the fifth cycle. In the area of oxygen efficiency, the TMS‐LiNO_3_ electrolyte was far better than other electrolytes (Figure 7l). The TMS electrolyte showed an oxygen efficiency at charge (η_O2_, _ch_) of as much as 81% in the first cycle, which was 10–20% higher than the other electrolytes (68%, 59%, and 74% for DMA, DMSO, and TEGDME, respectively). In addition, the gap in the oxygen efficiency became wider as the cycling proceeded. The TMS‐LiNO_3_ electrolyte achieved an 83% oxygen efficiency at the fifth cycle, while the DMA, DMSO and TEGDME electrolytes exhibited only 62%, 53%, and 64%, respectively.

**Figure 8:**
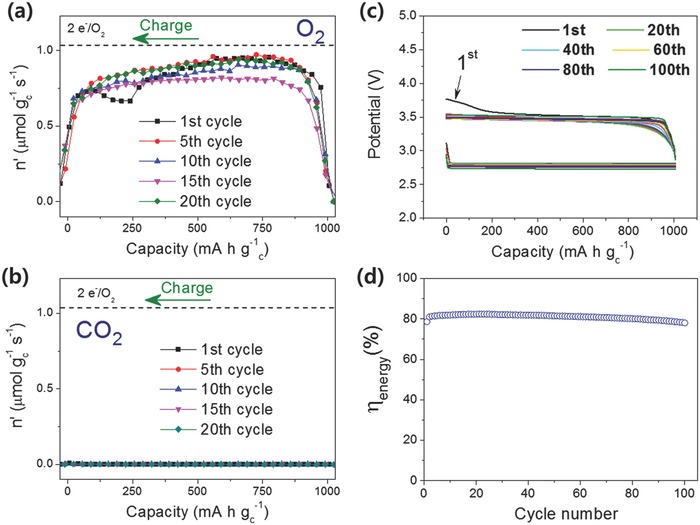
a) O_2_ evolution rate and b) CO_2_ evolution rate at charge in Li‐O_2_ cells with TMS electrolyte containing 1 m LiNO_3_ during the 20‐cycle test. c) Potential profiles and d) energy efficiency during 100 cycles of the Li‐O_2_ cell with TMS‐LiNO_3_ electrolyte.

In order to investigate the long‐term cycle performance of the TMS‐LiNO_3_ electrolyte, its oxygen efficiency and CO_2_ gas evolution were measured over 20 cycles using DEMS. Although there was some fluctuation, the oxygen efficiency at charge maintained a relatively stable value at around 80% during the 20 cycles (**Figure** **8**a and Figure S9a, Supporting Information). Moreover, a negligible amount of CO_2_ gas was observed over the long‐term cycling (Figure 8b and Figure S9b, Supporting Information), implying that the oxidative parasitic reaction was highly suppressed. Figure 8c,d and Figure S10 (Supporting Information) illustrate the potential profiles and energy efficiency during 100 cycles, as well as change in the impedance spectra for 100 cycles and the evolution behavior of O_2_ and CO_2_ gases at the 102^nd^ cycle. During 100 cycles, the Li‐O_2_ cell in the TMS‐LiNO_3_ electrolyte maintained a charge potential of about 3.5 V and an energy efficiency of about 80%, which were indicative of a stable NO_2_^−^/NO_2_ redox shuttle reaction present in the long‐term cycle test. There was not much change in the impedance from the first cycle to the 102^nd^ cycle, suggesting that the cell maintained stable cycling behavior with little parasitic reactivity. This long‐term cycling of the TMS‐LiNO_3_ electrolyte outperformed DMA‐LiNO_3_ electrolyte as shown in Figure S11 (Supporting Information). The Li‐O_2_ cell with the DMA electrolyte showed an abrupt capacity fade after just 20 cycles, which may have been due to continuous parasitic reactions and the accumulation of byproducts. The DEMS results in Figure S10b (Supporting Information) clearly show that the Li‐O_2_ cell maintained a stable oxidation of Li_2_O_2_ at charge achieving a charge oxygen efficiency of 80% and, moreover, the cell evolved almost no CO_2_ gas even at the 102^nd^ cycle. All these results demonstrated that the TMS‐LiNO_3_ electrolyte sustained superior cycle performance for more than 100 cycles.

The Li‐O_2_ cell with the TMS‐LiNO_3_ electrolyte achieved a full capacity of about 6000 mA h g_c_^−1^ at a current density of 300 mA g_c_^−1^, which required more than 40 h for discharge and charge cycling as shown in Figure S12a (Supporting Information). There was a noticeable plateau at 3.5 V during the charge profile at full depth of discharge, which was indicative of good operation of the NO_2_^−^/NO_2_ redox shuttle, similar to that at the controlled capacity of 1000 mA h g_c_^−1^ as shown in Figure 3d. The oxygen efficiency at charge was as high as 88%, which surpassed the efficiency at 1000 mA h g_c_^−1^, together with a very low amount of CO_2_ gas (*r*_CO2_ < 0.3%). The scanning electron microscopy (SEM) image and X‐ray diffraction (XRD) data in Figure S12c,d (Supporting Information) revealed that the discharge product was a crystalline Li_2_O_2_ with a toroidal shape with a diameter of about 0.5 µm. The toroidal Li_2_O_2_ particles deposited on the carbon cathode during the discharge were completely removed from the cathode surface following the charge process, as presented in SEM images in Figure S13 (Supporting Information) and XRD peaks in Figure S12d (Supporting Information). It is noteworthy that the large particles of Li_2_O_2_ are indicative of dominant occurrence of solution mechanism during the discharge process.^6^, ^36^ Such solution mechanism which takes place in the TMS‐LiNO_3_ electrolyte is favorable for enhancing discharge capacity.

## 3 Conclusion

In this work, as a result of a thorough evaluation and careful analysis, it is concluded that LiNO_3_ is very effective in reducing the charge overpotential of a Li‐O_2_ battery through a NO_2_^−^/NO_2_ redox couple that suppresses parasitic side reactions, producing long‐term reversible cycling. In addition, TMS was found to be the optimum solvent making effective use of the LiNO_3_ salt in an electrolyte compared to other typical solvents such as DMA, DMSO, and TEGDME. A detailed investigation using LSV‐DEMS analysis revealed that the TMS‐LiNO_3_ electrolyte effectively produces NO_2_^−^, which initiates the NO_2_^−^/NO_2_ redox reaction. The high compatibility of TMS with lithium metal was assumed to play a key role in the effective generation of NO_2_^−^, compared to DMA and DMSO. The high dielectric constant of TMS is yet another advantage of this solvent over TEGDME, which helped to facilitate the generation of NO_2_^−^ in the electrolyte. The low donicity of TMS allowed a lower redox potential of NO_2_^−^/NO_2_ couples and a reduced potential at the charging process. Overall, TMS appeared to be highly favorable for fostering an efficient NO_2_^−^/NO_2_ redox reaction, which produced a very low charge overpotential in the Li‐O_2_ cell, suppressed CO_2_ evolution, improved the oxygen efficiency and energy efficiency of the cell, and promoted a stable, long‐term cycle behavior for more than 100 cycles.

## 4 Experimental Section

*Preparation of Li‐O_2_ Cells*: Coin‐type Li‐O_2_ cells was composed of a Li metal anode, a glass microfiber membrane separator, and a Ketjen black (KB, EC‐600JD) cathode. In the cycling test system, high‐purity oxygen gas (>99.999%) was fed through the inlet capillary attached to the upper side of the cell holder into the KB cathode. In a typical Li‐O_2_ cell cycle, a current of 200 mA g_c_^−1^ (g_c_: weight of KB in the cathode) was applied for 5 h for both the discharging and charging of the cell with a cutoff potential of 2.0 V for the discharge and 5.0 V for the charge. All the potentials reported in this paper were displayed relative to the voltage of the Li/Li^+^ couple, unless otherwise stated.

*In Situ DEMS Analysis*: The consumption of O_2_ during the cell discharge and evolution of O_2_ and other gaseous products during the cell charge were quantitatively measured using in situ DEMS analysis. The details of the experimental apparatus and the operation of the in situ DEMS for the Li‐O_2_ cells have been reported in our previous publications.^20^, ^21^, ^22^, ^27^, ^37^

*LSV‐DEMS Analysis*: The Li‐O_2_ cells containing a Li metal anode, an electrolyte‐soaked separator, and the KB cathode were assembled in an Ar‐filled glove box and then used for LSV‐DEMS analysis according to the methods reported in our previous publications.^20^, ^21^ The cell potential was swept linearly from the OCV to 5.0 V at a scan rate of 0.1 mV s^−1^.

## Conflict of Interest

The authors declare no conflict of interest.

## Supporting information

Supplementary
