# Supplementary material for: High‐Performance Lithium‐Oxygen Battery Electrolyte Derived from Optimum Combination of Solvent and Lithium Salt
Source: Adv Sci (Weinh). 2017 Jul 25;4(10):1700235. doi: 10.1002/advs.201700235 (PMC5644260; doi:10.1002/advs.201700235)
Supplement: Supplementary file 1 — Supplementary [file ADVS-4-na-s001.pdf]

Copyright WILEY-VCH Verlag GmbH & Co. KGaA, 69469 Weinheim, Germany, 2017.

ADVANCED SCIENCE
Open Access

Supporting Information

for *Adv. Sci.,* DOI: 10.1002/advs.201700235

High-Performance Lithium-Oxygen Battery Electrolyte Derived from Optimum Combination of Solvent and Lithium Salt

Su Mi Ahn, Jungdon Suk, Do Youb Kim, Yongku Kang, Hwan Kyu Kim, and Dong Wook Kim**

Supporting Information

High-Performance Lithium Oxygen Battery Electrolyte Derived from Optimum Combination of Solvent and Lithium Salt

*Su Mi Ahn, Jungdon Suk, Do Youb Kim, Yongku Kang *, Hwan Kyu Kim and Dong Wook Kim**

S. M. Ahn, Dr. J. Suk, Dr. D. Y. Kim, Dr. Y. Kang, Dr. D. W. Kim

Advanced Materials Division, Korea Research Institute of Chemical Technology, 141 Gajeong-ro, Yuseong-gu, Daejeon, South Korea

E-mail: dongwook@krict.re.kr, ykang@krict.re.kr

Prof. H. K. Kim

Global GET-Future Laboratory & Department of Advanced Materials Chemistry, Korea University, 2511 Sejong-ro, Jochiwon, Sejong 339-700, South Korea

Preparation of electrolytes

Tetramethylene sulfone (TMS, 99%), tetraethyleneglycol dimethylether (TEGDME, >99%), dimethylacetamide (DMA, 99.8%), and dimethylsulfoxide (DMSO, 99.5%) were purchased from Sigma-Aldrich, and all were purified by a vacuum distillation and then dried by addition of freshly activated 4 Å molecular sieves. Lithium bis(trifluoromethane) sulfonimide (LiTFSI, 99.95%), lithium trifluoromethane sulfonate (LiTf, 99.995%), lithium perchlorate ($LiClO_4$, 99.99%), and lithium nitrate ($LiNO_3$, 99.99%) were obtained from Sigma-Aldrich and dried at 150 °C under vacuum for two days. Sodium nitrite ($NaNO_2$, 99.999%, Sigma-Aldrich) was dried at 120 °C in a vacuum oven for two days. The water content of the solvent and the electrolytes was controlled to less than 20 ppm, which was determined by Karl Fischer titration (C30, Mettler Toledo). All of the electrolytes used in the Li-O_2 cell experiments were prepared by dissolving the appropriate quantity of each dried lithium salt in each anhydrous solvent to produce a solution with a salt concentration of 1 M with the exception of the TEGDME-$LiNO_3$ electrolyte. Due to its limited solubility, sufficient $LiNO_3$ was dissolved in the anhydrous TEGDME to produce a 0.5 M solution. Addition of a small quantity (~50 ppm) of water to the TEGDME will readily dissolve the $LiNO_3$ salt up to a concentration of 1 M.

Preparation of Li-O_2 cells

To prepare anodes for the cell, lithium metal foil (0.3 mm thick, Honjo Metal Co.) was cut into 14 mm diameter disks using a metal punch. A glass microfiber membrane (GF/C^{TM}, Whatman Co.) was dried at 150 °C under vacuum for two days before being used as a separator in the cell. Ketjen black® (KB, EC-600JD) was provided by AkzoNobel Chemical Co. and polytetrafluoroethylene (PTFE) powder was obtained

from Sigma Aldrich. Ketjen black® and PTFE were combined with an isopropyl alcohol/water mixture to produce a slurry. This slurry was coated onto a gas diffusion layer (TGP-H030, Toray Co.) and dried at 150 °C under vacuum to produce the cell cathode. The typical loading of the KB in the cathode electrode was ~0.5 mg/cm^2.

Coin-type Li-O_2 cells with a top SUS mesh were assembled in an argon-filled glove box (MBraun, H_2O & O_2 <1 ppm) for subsequent battery experiments. The Li-O_2 cell was composed of a Li metal anode, a glass microfiber membrane separator, and a KB cathode. The coin cell was inserted in a cell holder with two attached SUS capillaries (outside diameter: 1/16 inch) for gas to flow in and out of the cell. The cell holder with a Li-O_2 coin test cell was transferred from the glove box to a battery cycling test system with gas capillaries tightly capped. In the cycling test system, high-purity oxygen gas (>99.999%) was fed through the inlet capillary attached to the upper side of the cell holder into the KB cathode. The gas exited from the cathode through a second capillary (outlet) that allowed the exhausted oxygen to flow from the cell. The oxygen pressure on the cathode was maintained at ~1.5 bar during the cell cycling, with the inlet capillary kept open and the outlet closed. The cycling of the Li-O_2 cells was controlled by a VMP3 potentiostat (Biologic Science Instrument). In a typical Li-O_2 cell cycle, a current of 200 mA g_c^{-1} (g_c: weight of KB in the cathode) was applied for 5 h for both the discharging and charging of the cell with a cut-off potential of 2.0 V for the discharge and 5.0 V for the charge. All the potentials reported in this paper were displayed relative to the voltage of the Li/Li$^+$ couple, unless otherwise stated.

In situ differential electrochemical mass spectrometry (DEMS) analysis

The consumption of O_2 during the cell discharge, and evolution of O_2 and other gaseous products during the cell charge were quantitatively measured using in situ DEMS analysis. The pressure drop in the hermetically sealed Li-O_2 cell during

discharge was recorded to quantitatively measure the quantity of oxygen consumed. Following discharge, the oxygen in the cell was flushed out and replaced with argon. During the charging process, any evolved gases in the isolated cell were accumulated during a programmed interval (e.g., 10 min) and were then transferred by argon carrier gas into the mass spectrometer to identify and quantify the gases. The quantity of the gases was determined by comparing the intensity of the peaks of the gases recorded by the mass spectrometer analysis to the intensity of MS peaks generated by carrier gas argon.

Several of the parameters measured by the DEMS analysis are defined as follows. The discharge oxygen efficiency ($\eta_{O2,\ dis}$) is the ratio of the quantity of O_2 consumed during cell discharge compared with the amount of O_2 consumed in an ideal discharge reaction as represented by, $2Li^+ + 2e^- + O_2 \rightarrow Li_2O_2$. The charge oxygen efficiency ($\eta_{O2,\ ch}$) is the ratio of the quantity of O_2 evolved during the charging of the cell compared with that in the ideal charge reaction, ($Li_2O_2 \rightarrow 2Li^+ + 2e^- + O_2$). The CO_2 gas ratio (r_{CO2}) is defined as the amount of CO_2 produced on charge normalized to the quantity of O_2 produced in the ideal charge reaction. Energy efficiency (η_{energy}) on cycling is defined by the energy (energy is the product of current-time and potential) produced during discharge to the energy consumed during charge.

Linear sweep voltammetry (LSV)-DEMS analysis

The Li-O_2 cells containing a Li metal anode, an electrolyte-soaked separator, and the KB cathode were assembled in an Ar-filled glove box and then used for LSV-DEMS analysis. The cell potential was swept linearly from the open-circuit voltage (OCV) to ~5.0 V at a scan rate of 0.1 mV s^{-1}. The current generated during the linear potential sweep was recorded by a potentiostat and, in addition, any gases evolved during the potential sweep were identified and in situ quantified using a mass spectrometer. The

results of LSV-DEMS analysis allowed for real-time monitoring of the anodic (oxidative) current, as well as that of any gases produced by the electrochemical reactions including, O_2, CO_2, H_2, and NO_2. Throughout this work, LSV-DEMS analysis was performed on two kinds of Li-O_2 cells: 1) pristine Li-O_2 cells assembled prior to any cycle testing, and 2) Li_2O_2-loaded Li-O_2 cells that had been discharged to produce a capacity of 1000 mA h g_c^{-1} (some Li-O_2 cells were stabilized after the first or second cycle, and hence, for a consistent comparison between the different cells, the Li-O_2 cells were loaded with Li_2O_2 by performing a third discharge process after two discharge-charge cycles).

Characterization methods

After discharge or full cycling, the cells were disassembled, and the cathode was washed with fresh acetonitrile to remove the electrolytes, and then dried in an Ar-filled glove box. To avoid exposure to air, the cathodes were packed under Ar atmosphere before being transferred to the scanning electron microscopy (SEM) chamber, or sealed with a polyimide tape for X-ray diffraction (XRD) measurements. The surface morphology of the cathode was observed by FE-SEM (Tescan Mira 3 LMU FEG, Philips). XRD analysis of the cathodes was performed using an Ultima diffractometer (Rigaku) with a graphite monochromatized Cu Kα source (λ = 1.5406 Å). The XRD patterns were indexed using the JCPDS database.

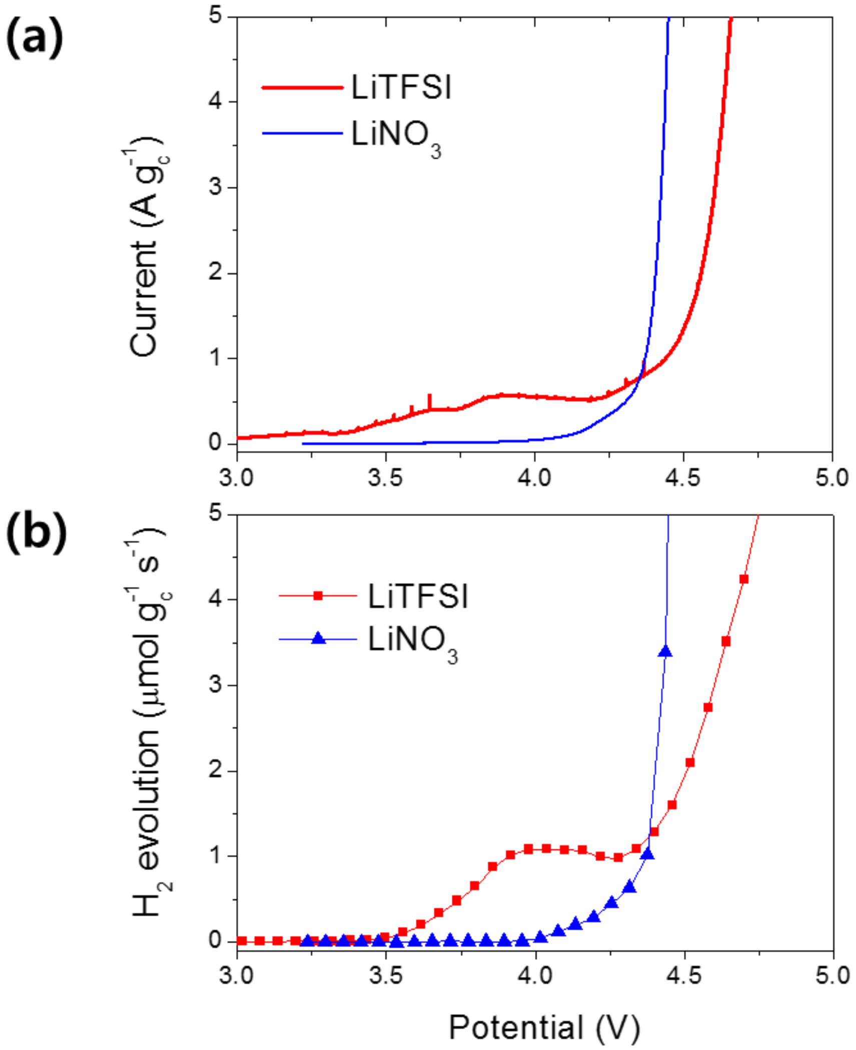

Figure S1. (a) Anodic current and (b) H_2 evolution rate measured by LSV-DEMS analysis during a linear oxidative scan from OCV to 5 V at a scan rate of 0.1 mV s^{-1}, which was performed on Li-O_2 cells with DMA-LiTFSI (red line) and DMA- $LiNO_3$ (blue line); lithium metal and KB-coated carbon paper were used as an anode and cathode, respectively.

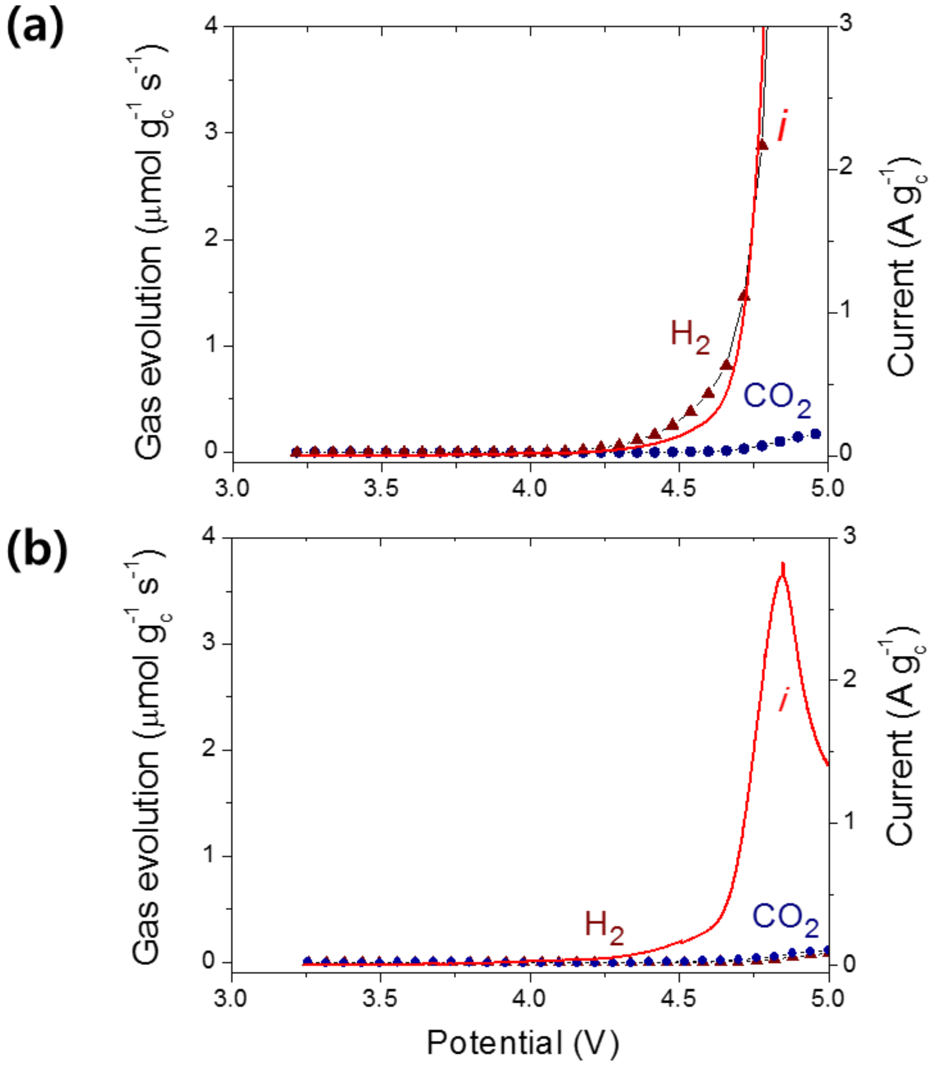

Figure S2. Anodic current (red line, right axis), CO_2 evolution rate (blue circle line, left axis), and H_2 evolution rate (red triangle line, left axis) observed by LSV-DEMS analysis, which was conducted on a Li-O_2 cell with TEGDME-LiTFSI electrolyte using (a) lithium metal and (b) lithium iron phosphate (LFP) as the anode. KB-coated carbon paper was used as a cathode. LFP was partially charged before use. The potential in the Li-O_2 cell with LFP was adjusted to the Li/Li^+ potential using 3.42 V for the LFP potential.

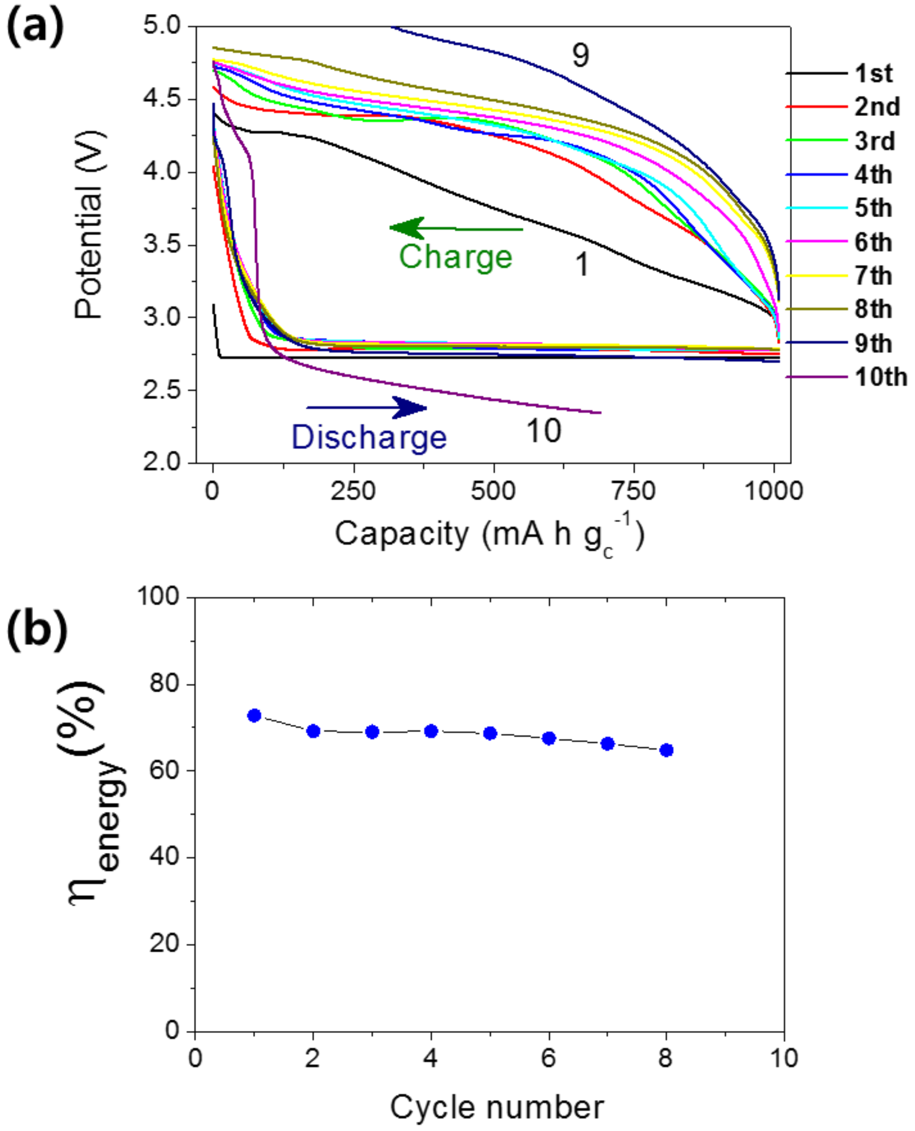

Figure S3. (a) Potential profiles and (a) energy efficiency of the Li-O_2 cells using TMS-LiTFSI electrolyte.

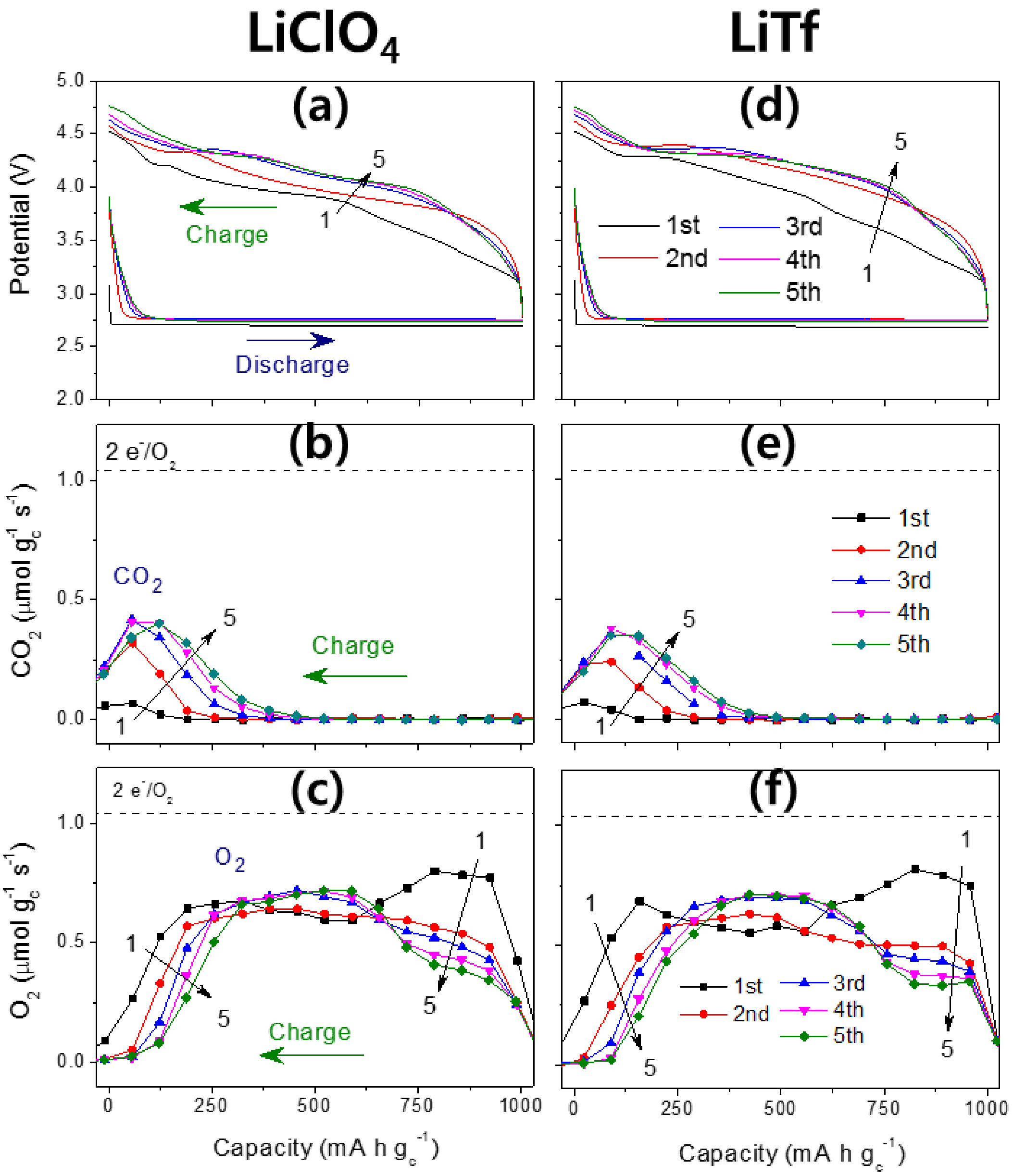

Figure S4. (a,d) Potential profiles, (b,e) CO_2 evolution rate at charge, and (c,f) O_2 evolution rate at charge in Li-O_2 cells with TMS electrolytes containing with (a,b,c) 1 M $LiClO_4$ and (d,e,f) 1 M LiTf over five cycles. The dashed lines labeled as 2 e^-/O_2 indicate the O_2 evolution rate following the ideal reaction of $2Li^+ + O_2 + 2e^- \leftrightarrow Li_2O_2$.

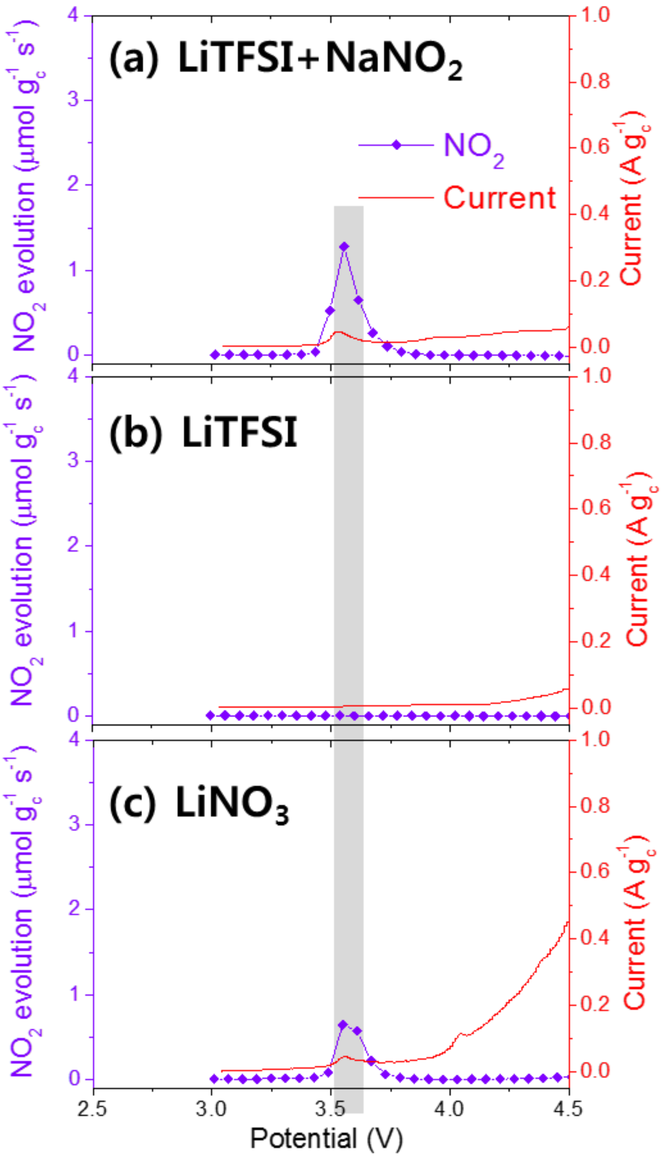

Figure S5. NO_2 evolution rate and anodic current measured by LSV-DEMS analysis during linear oxidative scan from OCV to 4.5 V at 0.1 mV s^{-1} conducted on pristine Li-O_2 cells using separate TMS electrolytes containing (a) 1 M LiTFSI and 20 mM $NaNO_2$, (b) 1 M LiTFSI, and (c) 1 M $LiNO_3$.

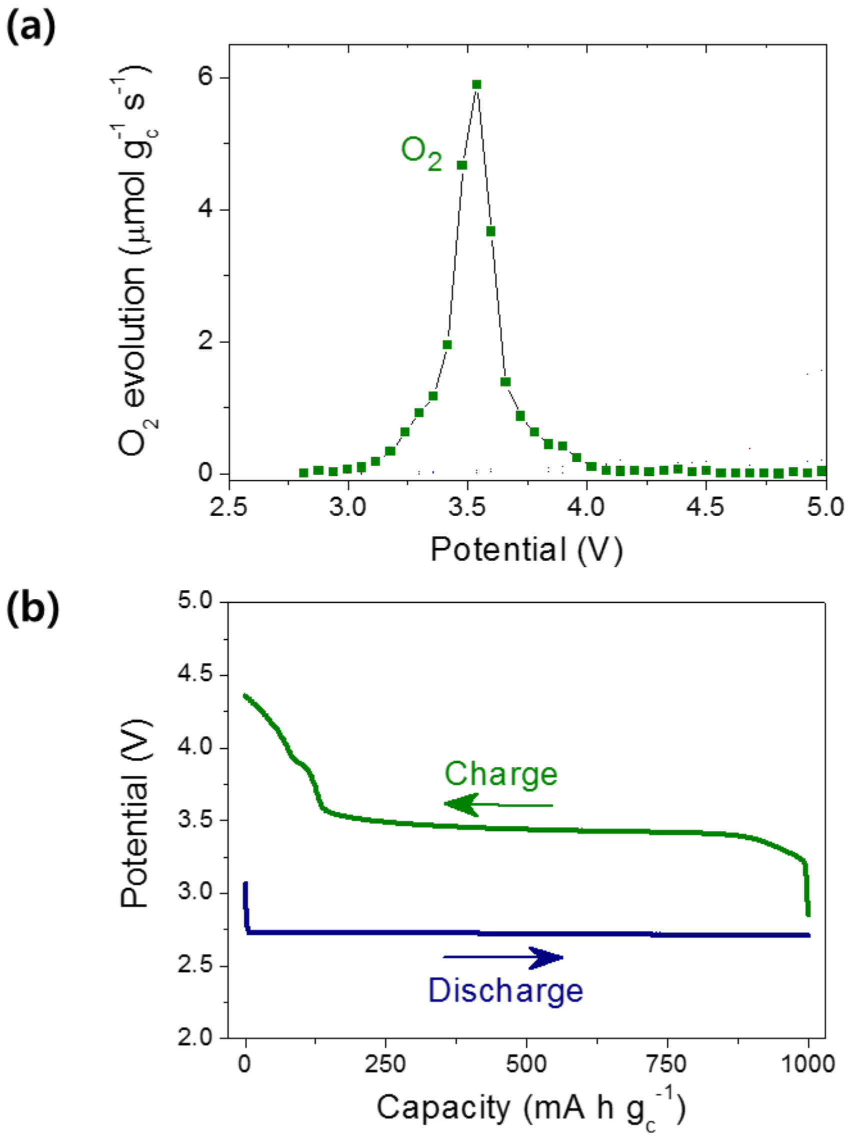

Figure S6. (a) O_2 evolution rate measured by LSV-DEMS analysis during linear oxidative scan from OCV to 5.0 V at a scan rate of 0.1 mV s^{-1}, which was conducted using a Li_2O_2-deposited Li-O_2 cell with TMS electrolyte containing 1.0 M LiTFSI and 20 mM $NaNO_2$. Li_2O_2 was in situ deposited on the Li-O_2 cells by performing a discharge process for a capacity of 1000 mA h g_c^{-1}. (b) potential profile of the Li-O_2 cells using TMS electrolyte containing 1.0 M LiTFSI and 20 mM $NaNO_2$ during the first cycle with a capacity of 1000 mA h g_c^{-1}.

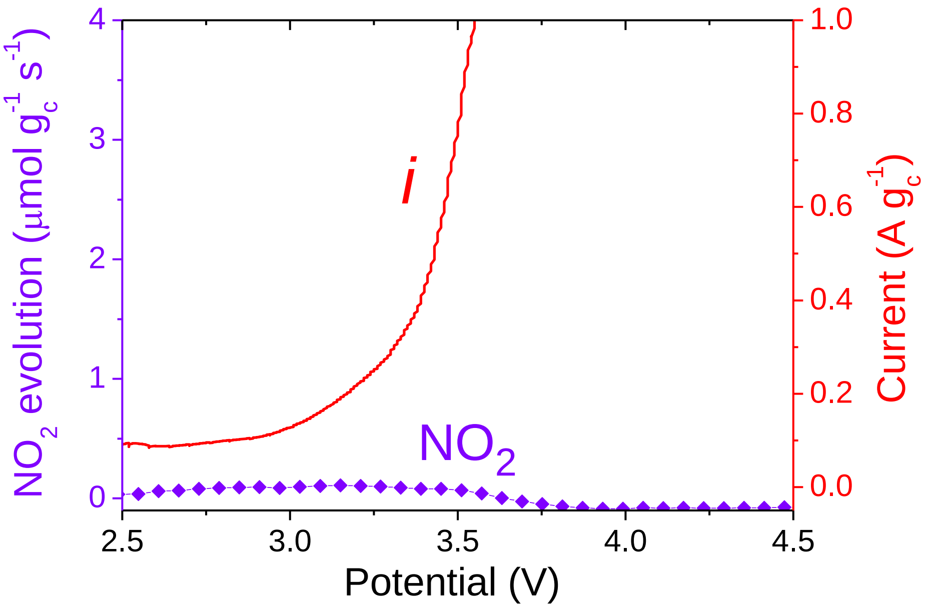

Figure S7. NO_2 evolution rate and anodic current measured by LSV-DEMS analysis during linear oxidative scan from OCV to 4.5 V at a scan rate of 0.1 mV s^{-1}, conducted on a Li-O_2 cells with a DMA electrolyte containing 1 M LiTFSI and 20 mM $NaNO_2$. Lithium metal and KB-coated carbon paper were used as the anode and cathode, respectively.

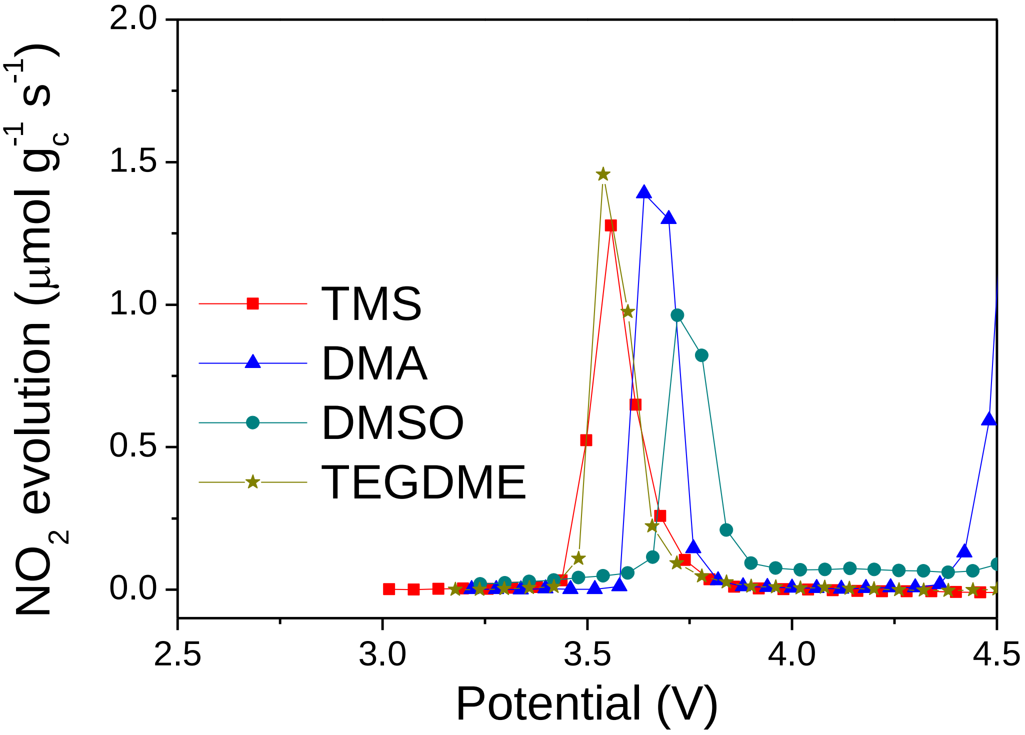

Figure S8. NO_2 evolution rate measured by LSV-DEMS in linear oxidative scan from OCV to 4.5 V at a scan rate of 0.1 mV s^{-1}, conducted on a Li-O_2 cell containing TMS, DMSO, and TEGDME electrolytes with 1 M LiTFSI and 20 mM $NaNO_2$, and DMA electrolyte containing 1 M $LiNO_3$ and 20 mM $NaNO_2$ the data were taken from Figures 5a-d.

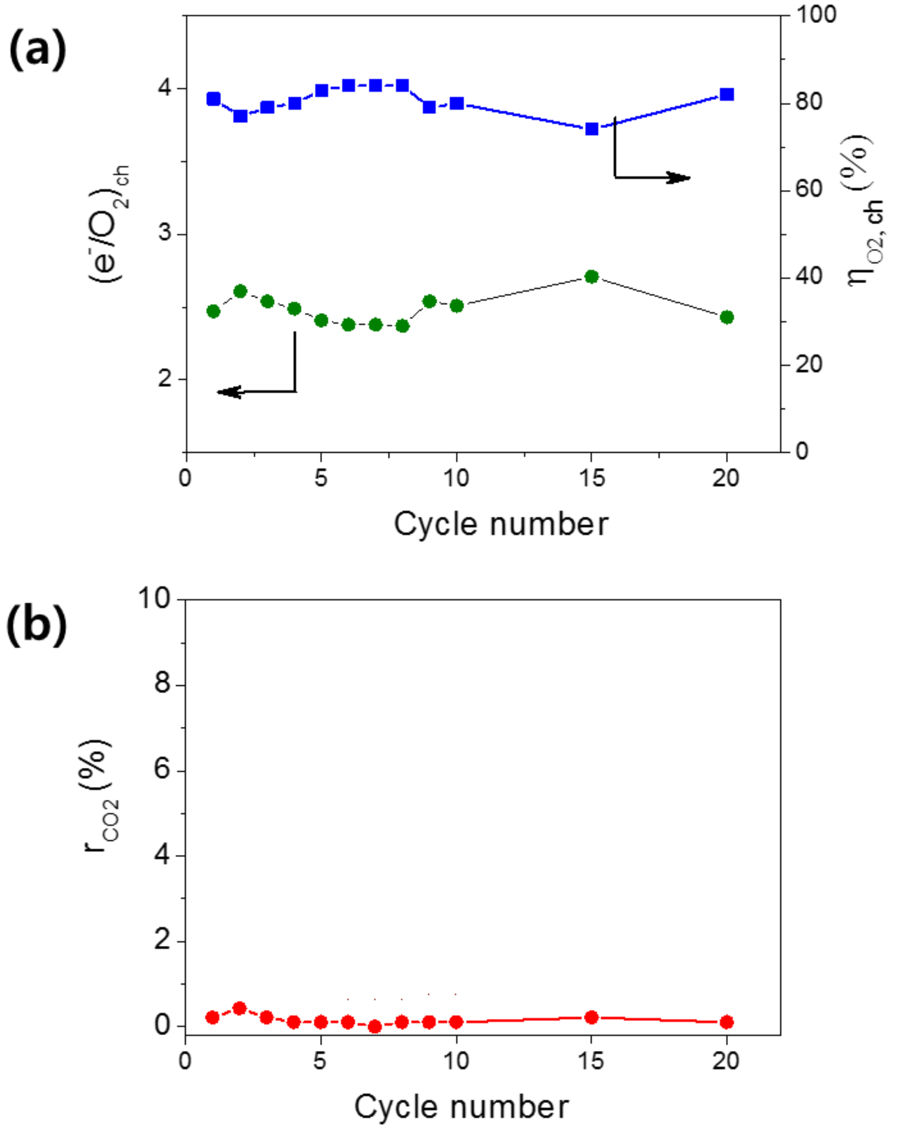

Figure S9. (a) Oxygen efficiency and e^-/O_2 ratio at charge and (b) CO_2 evolution ratio at charge in Li-O_2 cells with TMS electrolyte containing 1 M $LiNO_3$ during the 20-cycle test.

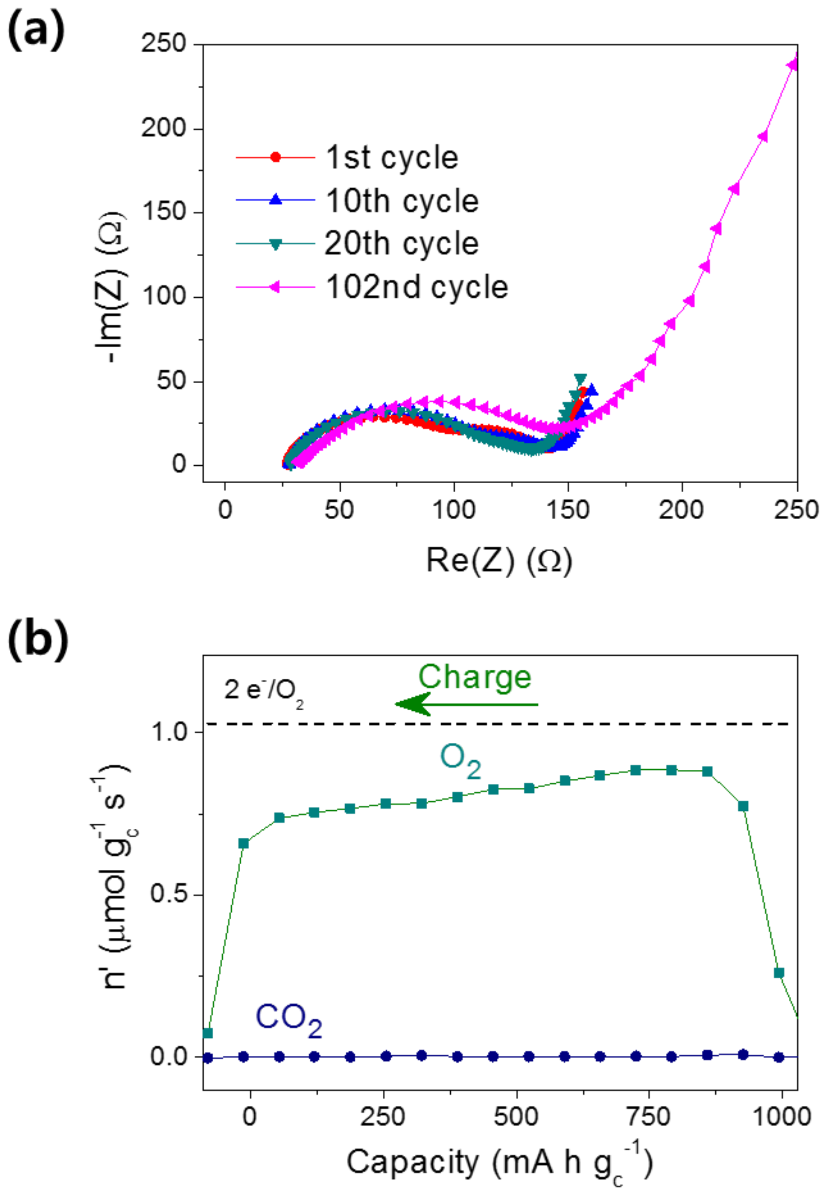

Figure S10. (a) Impedance spectra at the 1st, 10th, 20th, and 102nd cycle and (b) evolution rate of O_2 and CO_2 gases at the 102nd cycle of the Li-O_2 cell with TMS electrolyte containing 1 M $LiNO_3$.

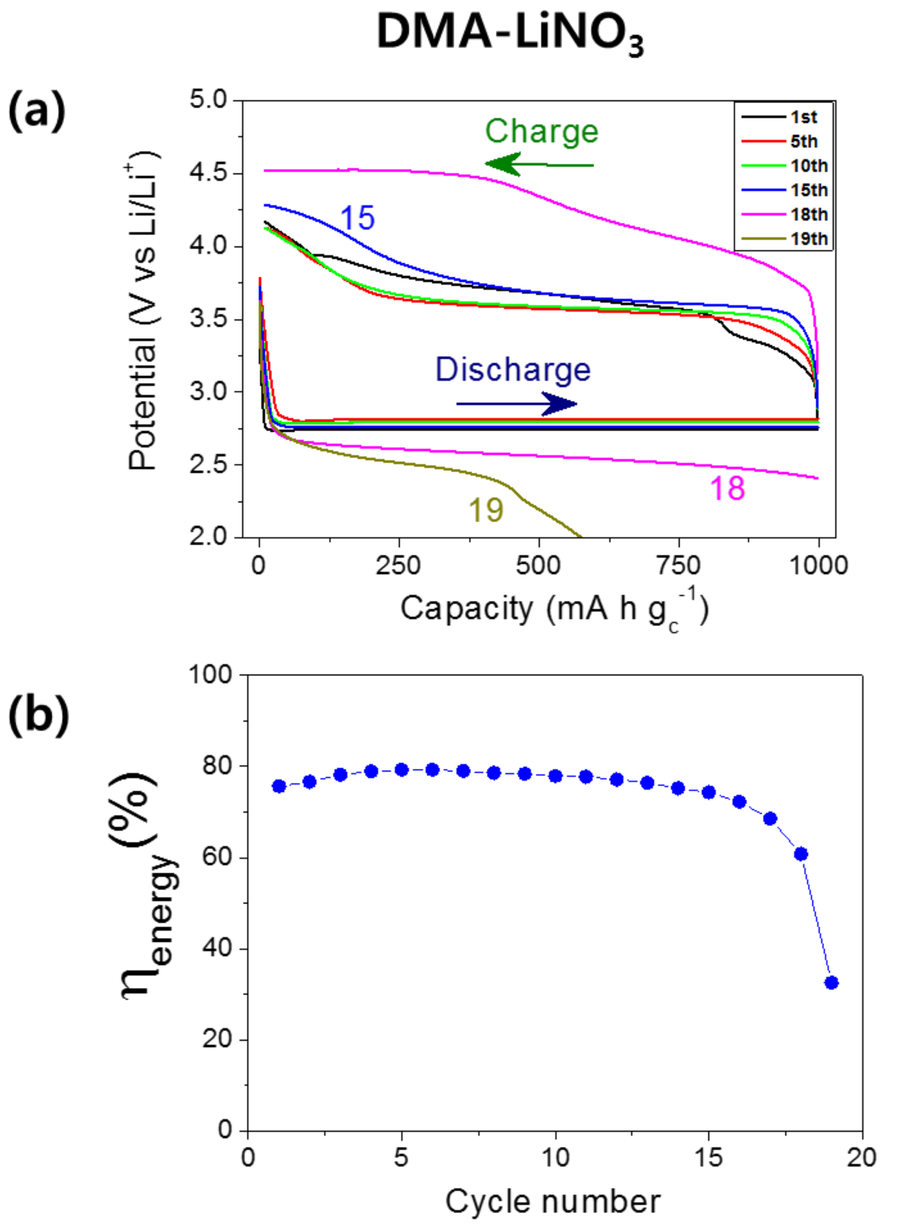

Figure S11. (a) Potential profiles and (b) energy efficiency of the Li-O_2 cells using a DMA-$LiNO_3$ electrolyte, 200 mA g_c^{-1} of current was applied to attain a cell capacity of 1000 mA h g_c^{-1} with a cut-off potential at 2.0 V during discharge and 5.0 V during charge.

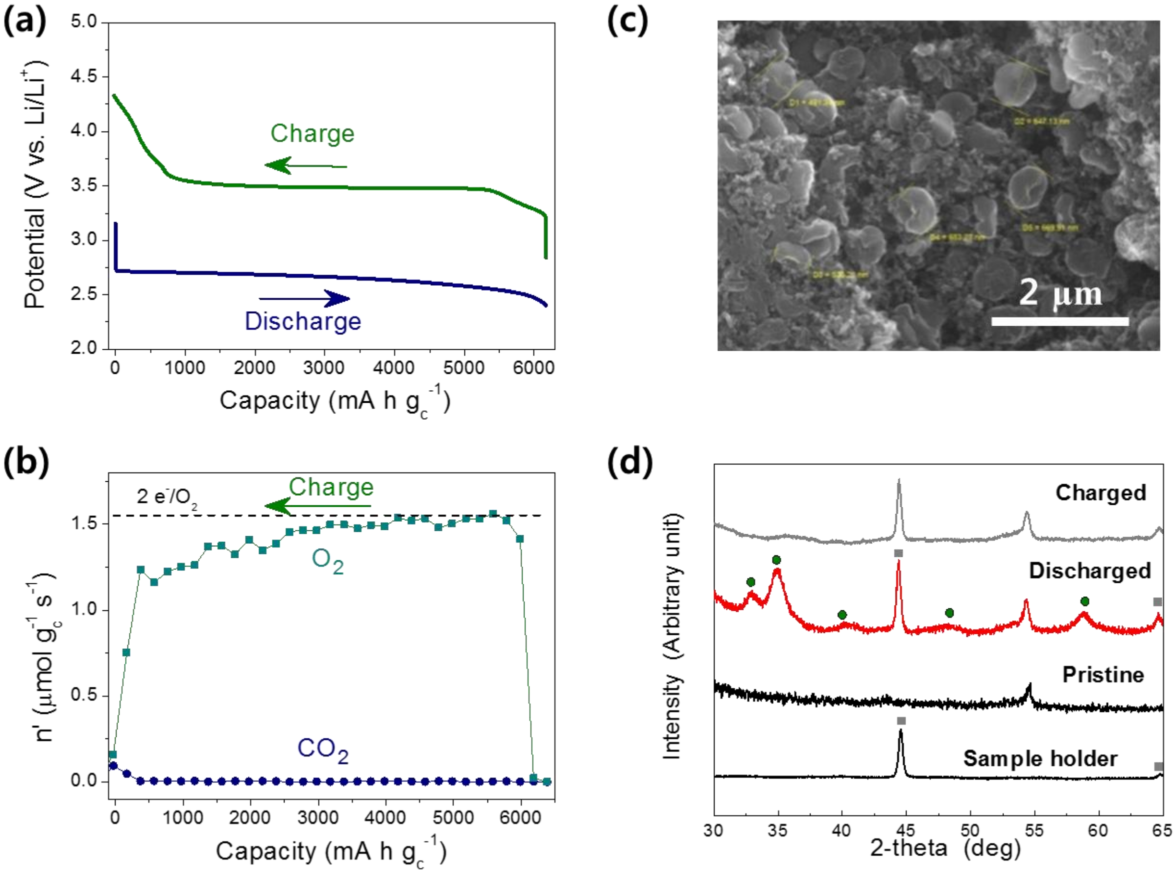

Figure S12. (a) Potential profile and (b) evolution rate of O_2 and CO_2 at charge in the 1st cycle at full depth of discharge (DOD), (c) SEM image of the discharged cathode, (d) XRD patterns of the pristine, discharged, and charged cathode together with an aluminum sample holder. The discharged and charged cathodes in (c) and (d) were taken from the Li-O_2 cells that employed TMS-1M $LiNO_3$ electrolyte after discharge and charge at full DOD as shown in (a). The green circles on the XRD peaks in (d) correspond to the peaks of crystalline Li_2O_2 and the square dot on the XRD peaks in (d) indicates the interference peak attributed to the sample holder.

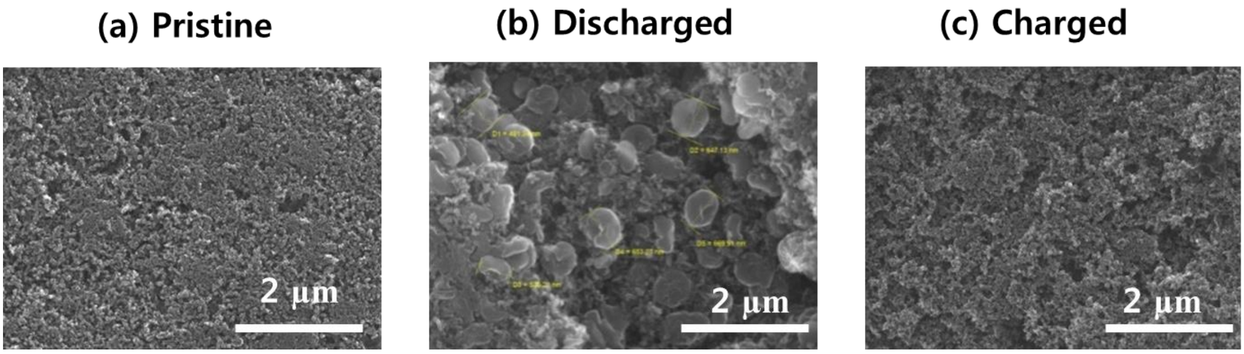

Figure S13. SEM images of the (a) pristine KB cathode, (b) discharged cathode, and (c) charged cathode; the discharged and charged cathodes in (b) and (c) were taken from the Li-O_2 cells containing a TMS-$LiNO_3$ electrolyte after discharge and charge at full DOD as shown in Figure S12a.
